# New spider flies from the Neotropical Region (Diptera, Acroceridae) with a key to New World genera

**DOI:** 10.3897/zookeys.270.4476

**Published:** 2013-02-18

**Authors:** Evert I. Schlinger, Jessica P. Gillung, Christopher J. Borkent

**Affiliations:** 1 c/o California Academy of Sciences, San Francisco, CA, 94118 USA; 2 Universidade de São Paulo, Departamento de Zoologia, Instituto de Biociências, São Paulo, SP, Brazil; 3 California State Collection of Arthropods, California Department of Food and Agriculture, 3294 Meadowview Road, Sacramento, CA 95832, USA

**Keywords:** Acrocerinae, Panopinae, Philopotinae, parasitoid, small-headed flies

## Abstract

Two new genera and five new species of spider flies (Diptera: Acroceridae) are described from the Neotropical Region. A new genus of Philopotinae (*Neophilopota brevirostris* Schlinger **gen. et sp. n.**) is described from Mexico, while an unusual new species of *Sphaerops* Philippi, 1865 (Acrocerinae: *Sphaerops micella* Schlinger **sp. n.**) is described from Chile. A new Panopinae genus near *Lasia* Wiedemann, 1824 (*Coquena stangei* Schlinger **gen. et sp. n.**), is described from Argentina and two new species of *Pialea* Erichson, 1840 (*Pialea brunea* Schlinger **sp. n.** and *Pialea corbiculata* Schlinger **sp. n.**)are described from Venezuela. Each genus is diagnosed and figured, and a key to species provided. The Neotropical fauna presently includes 19 genera, containing approximately 100 species. A key to New World genera is also included.

## Introduction

Spiders flies (Diptera, Acroceridae), also known as small-headed flies, are a family of infrequently collected lower brachyceran flies. The sister family is thought to be the Nemestrinidae, and these two families are sometimes united as the Nemestrinoidea, or are considered the sister lineages to the remainder of the Muscomorpha (see further discussion in Woodley et al. 2009). The family is widespread geographically, with species found in all biogeographical regions. Species are very diverse in size, shape and coloration, but they typically present a small head, greatly enlarged lower calypter and swollen abdomen (Schlinger 1981, 1987). Some species feed at flowers, and may be specialized pollinators, as suggested by their long proboscises (often equal to their body length), nectar feeding habits and pollen loads (Borkent and Schlinger 2008a, b). All species with known immature habits are parasitoids of spiders (Schlinger 1981, 1987; Cady et al. 1993; Larrivée and Borkent 2009).

Acroceridae comprise approximately 520 species in 53 genera (Pape et al. 2011; Gillung and Winterton 2011; Winterton 2012; Winterton and Gillung 2012). The family is classified into three extant subfamilies (Panopinae, Acrocerinae, and Philopotinae) based on adult morphology and host preference. Panopinae is considered by some authors as the sister group to the remaining Acroceridae (Schlinger 1987, 2009). However, phylogenetic analyses using DNA sequence data disagree with this subfamilial arrangement, and suggest that Acrocerinae are polyphyletic, due to placement of the Philopotinae in between two groups of Acrocerinae, and Panopinae are instead a derived clade (Winterton et al. 2007).

The Neotropical spider fly fauna includes all three subfamilies and is represented by 19 genera and approximately 100 species. Five philopotine genera are recorded for the Neotropics: *Megalybus* Philippi, 1865 (Chile); *Terphis* Erichson, 1840 (Brazil); *Quasi* Gillung and Winterton, 2011 (Mexico); *Philopota* Wiedemann, 1830, (Neotropical) and the new genus *Neophilopota* gen. n. *Neophilopota* is described here from Mexico and appears closely related to *Philopota*.

Panopinae is the most diverse subfamily, represented by ten genera in the New World. Six of these are endemic to South America - *Archipialea* Schlinger, 1973; *Camposella* Cole, 1919; *Exetasis* Walker, 1852; *Lasioides* Gil Collado, 1928; *Coquena* gen. n. Schlinger; *Pialea* Erichson, 1840; and *Pteropexus* Macquart, 1846. *Coquena* is described from Argentina and appears closely related to *Lasia*, based on head and wing characters. *Apelleia* Bellardi, 1862 is apparently endemic to Central America, *Lasia* Wiedemann, 1824 and *Ocnaea* Erichson, 1840 are distributed from Central to South America, and *Eulonchus* Gerstaecker, 1856 is restricted to North America.

Acrocerinae is represented in the Neotropical Region by six genera, three of them, *Sphaerops* Philippi, 1865; *Villalus* Cole, 1918; and *Holops* Philippi 1865, are endemic to Chile, while *Pterodontia* Gray, 1832; *Ogcodes* Latreille, 1797; and *Acrocera* Meigen, 1803 are largely cosmopolitan (Cole 1919; Schlinger 1981, 2009). *Sphaerops* is a monotypic genus described by Philippi from Chile. The only included species, *Sphaerops appendiculata* Philippi, is remarkable in its biology. While most acrocerid larvae are internal parasitoids of spiders, larvae of *Sphaerops appendiculata* are suggested to be external parasitoids (Schlinger 1987). Winterton et al. (2007) placed *Sphaerops* with *Acrocera* in a clade that is sister to the rest of Acroceridae, while the remaining acrocerine genera sampled were recovered in a second polyphyletic ‘acrocerine’ clade sister to Panopinae.

In this manuscript we describe two new acrocerid genera (a philopotine and a panopine) and five new species. A complete key to all 21 genera occurring in the New World is provided.

## Material and Methods

Terminology follows Schlinger (1981) as modified by Cumming and Wood (2009), Gillung and Winterton (2011) and Winterton (2012). Type specimens are deposited in the California Academy of Sciences, San Francisco, CA, USA (CAS), and the National Museum of Natural History, Washington DC, USA (USNM). Specimen images were taken at different focal points using a digital camera and subsequently combined into a serial montage image using Helicon Focus software. All new nomenclatural acts and literature are registered in Zoobank (Pyle and Michel 2008).

Forty-three specimens were examined from the two collections listed above. The specimens were compared to previously published descriptions and figures and did not agree with any described species in the case of *Sphaerops* and *Pialea*, or described genera in the case of the two new genera. The exact label information for primary types is provided, with line breaks indicated with ‘/’ and handwriting in italics. Any inferred label information is in square brackets. Wing measurements were taken as the length from the base of the distal median plate to the wing tip. Body length was recorded as the distance from the anterior margin of the scutum to the posterior margin of abdominal segment VI when viewed dorsally. The holotype was always included in the series of measured specimens. Distribution maps were made using Simplemappr (Shorthouse 2010).

## Taxonomy

### Key to New World genera of Acroceridae

**Table:** 

1	Postpronotal lobes greatly enlarged, meeting or nearly meeting along midline to form collar for head; body shape strongly arched (Figs 6, 7)	Philopotinae: 3
–	Postpronotal lobes not greatly enlarged, separate along midline; body not strongly arched (Figs 3, 13)	2
2. (1)	Antenna with flagellum stylate (never longer than head length) (Figs 1, 3); tibiae without apical spines (except in *Pterodontia*, see couplet 12)	Acrocerinae: 7
–	Antenna with flagellum cylindrical, tapered, or flattened (usually longer than head length) (Fig. 11, 20); tibiae with apical spines	Panopinae: 13
3. (1)	Wing venation reduced, M with only one or two branches, and only basal cell (br) present (Fig. 10)	4
–	Wing venation relatively complete, all branches of M present as well as discal and r_4+5_ cells (South America)	*Megalybus*Philippi
4. (3)	Eyes bare	5
–	Eyes covered with short pile (Fig. 8)	6
5. (4)	Pairs of tubercles present on tergites II–IV of abdomen; occiput extended posteriorly to form an acute ridge (South America) (Fig. 25)	*Terphis* Erichson
–	Abdomen without tubercles; occiput rounded, not extended posteriorly (Gillung and Winterton 2011: figs 3–5) (Central America)	*Quasi* Gillung and Winterton
6. (4)	Frons well developed, almost twice as long as wide and longer than antennae; antennae inserted in the middle of frons; lower facial margin the same width in the upper and lateral portions; clypeus longer than antennae (Figs 26, 27)	*Philopota* Wiedemann
–	Frons not developed, as long as wide and shorter than antennae; antennae inserted in the lower part of frons, closer to mouthparts; lower facial margin wider in the upper portion than in lateral portions; clypeus shorter than antennae (Figs 8, 9)	*Neophilopota* Schlinger gen. n.
7. (2)	Cell m_3_ present and well defined (i.e., Fig. 28)	*Holops* Philippi
–	Cell m_3_ clearly absent (Fig. 10), *or*, fusion of m_3_ with discal cell indicated by presence of spur veins (rare)	8
8. (7)	Antennae located on upper half of head, usually proximal to frons (Fig. 3)	9
–	Antennae located on lower half of head, adjacent to mouthparts	12
9. (8)	Vein R_4+5_ present as a single, unforked vein originating along anterior margin at (or near) apex of cell r_4+5_ (Figs 2, 5); antennae not immediately adjacent to ocellar tubercle (rarely immediately adjacent)	10
–	Vein R_4+5_ originating at apex of basal cell r_4+5_ and then forking into veins R_4_ and R_5_ (i.e. Fig. 36); antennae always located on head immediately adjacent to ocellar tubercle	11
10. (9)	Eyes sparsely pilose, setae barely evident; wing veins A_1_ and CuA_2_ either separate (Fig. 2) or fusing near wing margin (Fig. 5); flagellum with minute terminal seta; genitalic capsule relatively enlarged and bulbous; body dark (Chile)	*Sphaerops* Philippi
–	Eyes densely pilose; A_1_ not joined to CuA_2_, either incomplete, or open to wing margin (Fig. 37); flagellum with relatively large terminal seta; genitalic capsule not enlarged or bulbous; body orange (Chile)	*Villalus* Cole
11. (9)	Wing with single medial vein; at most two wing cells present (br and bm); alula well developed (Schlinger 1981: figs 17–20) (Cosmopolitan)	*Acrocera* Meigen
–	Wing with three medial veins originating from discal cell; four wing cells present; alula reduced (Schlinger 1981: Figs 21–22) (Nearctic and Caribbean)	*Turbopsebius* Schlinger
12. (8)	Tibial spines present; mouthparts present (Cosmopolitan); wing with at least four closed cells (Fig. 29)	*Pterodontia*Gray
–	Tibial spines absent, mouthparts absent, buccal cavity closed; wing with at most two closed wing cells (Winterton 2012: fig. 3C) (Cosmopolitan)	*Ogcodes* Latreille
13. (2)	Pulvilli and empodium present (i.e. Cumming and Wood 2009: fig. 49); flagellum shape variable	14
–	Pulvilli and empodium absent; flagellum extremely large and paddle-like (South America, known only from male)	*Camposella* Cole
14. (13)	Mouthparts longer than head	15
–	Mouthparts shorter than head	18
15. (14)	Wing costal margin abruptly bent distally so that wing apex is truncated (i.e., Schlinger 1981: fig. 15) (South America)	*Pteropexus* Macquart
–	Wing costal margin uniform and continuous with rounded apex (i.e. Fig. 15)	16
16. (15)	Eyes contiguous below the antennae; palp present; alula absent (Nearctic)	*Eulonchus* Gerstaecker
–	Eyes separated below the antennae (i.e. Fig. 26); palp absent; alula present (Neotropical and Nearctic)	17
17. (16)	Antenna elongate, tapered cylinder, not strongly flattened; ocellar tubercle rarely raised (New World)	*Lasia* Wiedemann
–	Antenna strongly flattened laterally and paddle-like (i.e. Figs 11, 12); ocellar tubercle prominent (South America)	*Lasioides* Gil Collado
18. (14)	Scapes exhibiting total (Fig. 30) or partial (Fig. 31) fusion (South America)	*Pialea* Erichson
–	Scapes separate	19
19. (18)	Ocellar tubercle strongly raised (twice as high as wide), shaped like a crown (Fig. 13) (South America)	*Coquena* Schlinger gen. n.
–	Ocellar tubercle not strongly raised	20
20. (19)	Antennae inserted adjacent to ocellar tubercle (Neotropical and Nearctic)	21
–	Antennae inserted between the middle of frons and mouthparts (Central America)	*Archipialea* Schlinger
21. (20)	Eyes bare (Central America)	*Apelleia* Bellardi
–	Eyes pilose (i.e. Figs 30, 31)	22
22. (21)	Vein R_4_ absent (South America)	*Exetasis* Walker
–	Vein R_4_ present (i.e. Fig. 19) (Neotropical and Nearctic)	23
23. (22)	Eyes widely separated above antennae (Cole 1919: fig. 12a); ocellar tubercle raised (Neotropical and Nearctic)	*Ocnaea* Erichson
–	Eyes narrowly separated above antennae; ocellar tubercle at most slightly raised (Chile)	*Arrhynchus* Philippi.

### New Acroceridae from the Neotropical Region

Subfamily Acrocerinae Zetterstedt, 1837

#### Sphaerops

Philippi, 1865

http://species-id.net/wiki/Sphaerops

##### Diagnosis.

Body shape not arched; coloration non-metallic. Head width slightly less than thorax width; nearly spherical in shape; ocellar tubercle raised and rounded with three ocelli; postocular ridge and occiput rounded; posterior margin of eye rounded; eye sparsely pilose with minute setae (not more than 4× length of single ommatidium); eyes either contiguous above antennal base or with antennal base adjacent to dorsal eye margin, contiguous below antennal base; palpus absent; proboscis length greatly reduced with sparse pile; antennae located near or adjacent to ocellar tubercle; flagellum stylate, apex with terminal seta(e); scapes not fused together; postpronotal lobes not enlarged or contiguous medially; antepronotum narrow; subscutellum enlarged; legs not elongated; tibial spines absent; pulvilli present; wing markings and microtrichia absent. Costal vein ending near wing apex; costal margin straight; humeral crossvein absent; R_1_ inflated at pterostigma; radial veins straight, veins R_4_ and R_5_ present as single fused vein; crossvein 2r-m present between M_1_ and R_4+5_, bisecting cell r_4+5_, basal portion of cell narrow elongate; two M veins present, not reaching wing margin; discal cell closed; cell m_3_ absent; CuA_1_ joining M_3_; anal lobe well developed; alula well developed. Abdomen greatly rounded, inflated, tergites smooth.

##### Comments.

*Sphaerops* is an endemic Chilean genus than can be readily differentiated from all other acrocerine genera based on the sparsely pilose eyes, wing vein A_1_ fused to CuA_2_ and the bulbous genitalia. *Sphaerops* is also unique in exhibiting the widest range in size variation within acrocerids. The genus shows remarkable similarity to the Chilean endemic genus *Villalus*, sharing numerous characteristics such as having the antennae placed away from the ocellar tubercle (except in *Sphaerops micella*) and vein R_4+5_ present as a single, unforked, vein. Evert I. Schlinger has reared numerous *Sphaerops* individuals and reported that the mature larvae fed externally on spiders for up to three weeks. This type of development is unique as all other acrocerids with known larval habits are endoparasitoids until emerging to pupate (Schlinger 1987).

##### Key to species of Sphaerops

**Table:** 

1	Antennae immediately adjacent to ocellar tubercle; wing veins pale (Fig. 1); wing veins CuA_1_ and A_1_ not fused, cell cu-p absent (Fig. 2); smaller species (mean body length= 1.9 mm)	*Sphaerops micella* Schlinger sp. n.
–	Antennae not immediately adjacent to ocellar tubercle (Fig. 3); wing veins dark; CuA_1_ and A_1_ fused, cell cu-p present (Fig. 5); larger species (mean body length 6.3 mm)	*Sphaerops appendiculata* Philippi, 1865.

#### Sphaerops micella

Schlinger sp. n.

urn:lsid:zoobank.org:act:32AB0AE4-47D5-42C9-9BCC-2AC1CB4B281A

http://species-id.net/wiki/Sphaerops_micella

Figs 1 2 24

##### Material examined.

**Holotype** male: Top label: “CHILE, **Region III, Copiapó Prov** / 125 km SE Copiapó; Fundo La / Semilla; malaise on alluvium nr river; / 30.x-9.xi.2003; ME Irwin; FD Parker / 2358 m; 28°15.04'S, 69°44.46'W”. Bottom label: red “HOLOTYPE ♂/ *Sphaerops micella*/ Schlinger new species/ Det. E.I. Schlinger 2012” (CAS).

##### Paratypes.

All from Chile and bearing yellow paratype labels. Some with genitalia dissected and placed in glycerin in microvial on pin with specimen. Same data as holotype (4 ♂, CAS); Same except: 2 km E Puente La Semilla: 119 km SE Copiapó; malaise nr wash with water; 11–17.x.2003; ME Irwin; 2082 m; 28°12.90'S, 69°45.66'W (1 ♂, CAS); Quillota Province; Palma de Ocoa; Parque Nacional Campanas; malaise in hillside draw; 215 m; 29.xii.1999; ME Irwin, EI Schlinger; 32.9324°S, 71.0781°W (EI Schlinger #010913; 1 ♂, CAS); same except: 2–10.i.2000 (EI Schlinger #013443-013449; 7 ♂, CAS); Region VI, Limarí Prov. Frey Jorge Nat’l Park, Quebrada Honda I; malaise in small wash; 15–31.x.2003; ME Irwin, FD Parker; 122 m; 30°41.4'S, 71°37.8'W (8 ♂); same except: 1–7.xi.2003 (ME Irwin #174749-174752, #174754-174756; 6 ♂, 1?, CAS); same except: El Mineral, malaise in wash upstream of seep, 23.xi–12.xii.2003; ME Irwin, FD Parker; 224 m; 30°39.44'S, 71°39.90'W (2 ♂, CAS).

##### Diagnosis.

This species is much smaller than *Sphaerops appendiculata* (1.9 mm versus 6.3 mm) and has shorter pile on the thorax and abdomen. It is also unique in having the antennae placed immediately adjacent to the ocellar tubercle, the wing veins pale yellow, and wing veins CuA_2_ and A_1_ remaining separate.

##### Description.

**Figure 1.**
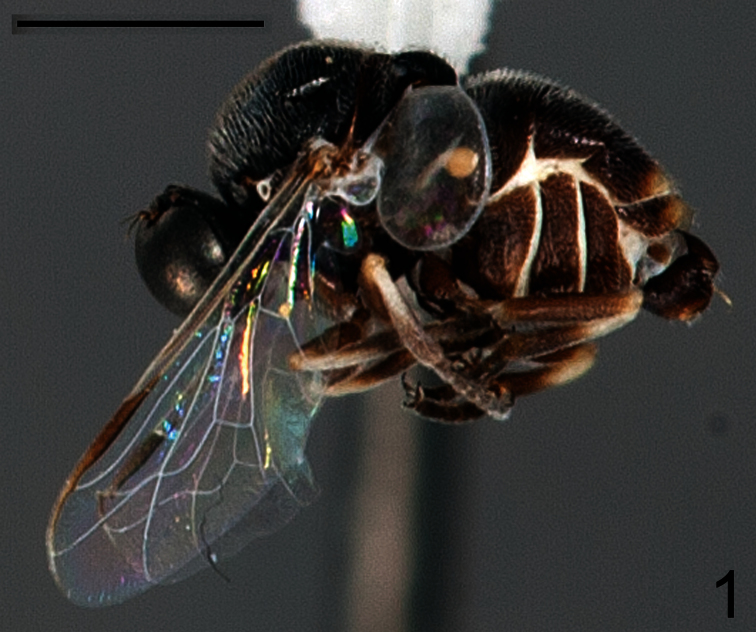
*Sphaerops micella* Schlinger sp. n., male paratype, lateral view. Scale bar = 1.0 mm.

**Figure 2.**
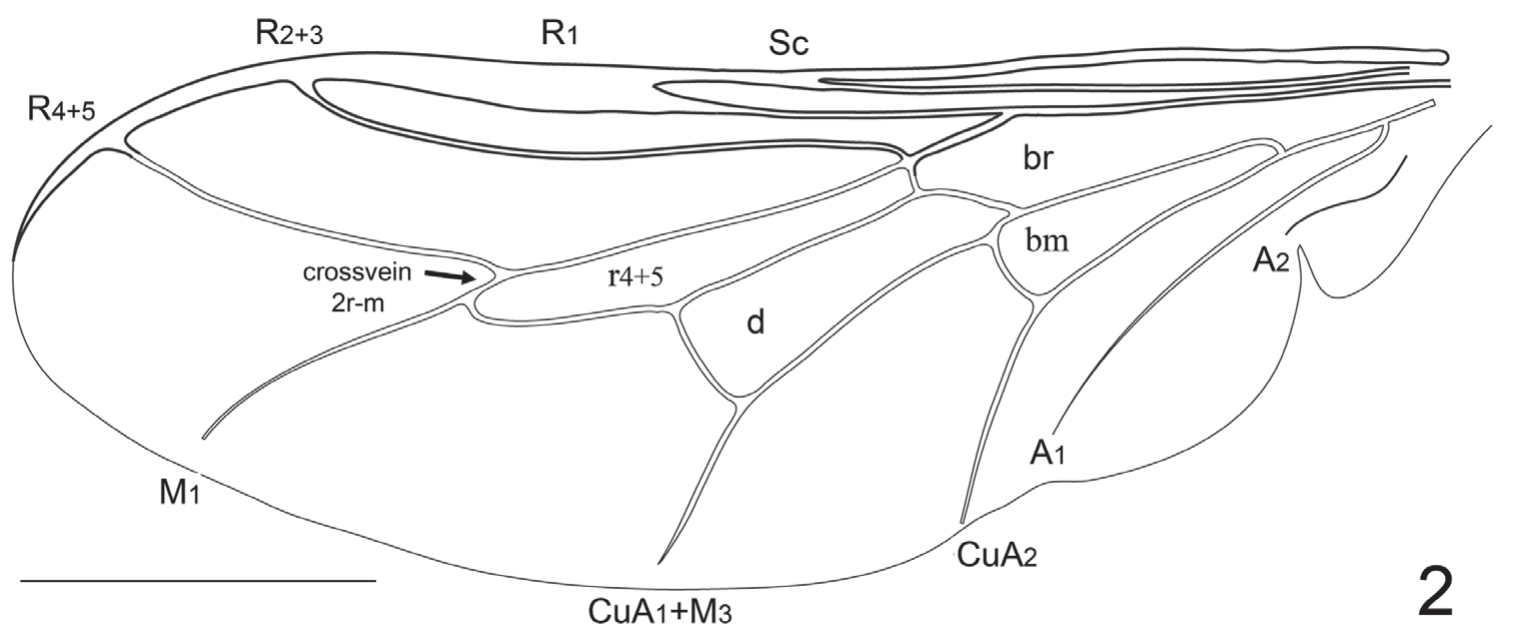
*Sphaerops micella* Schlinger sp. n., wing. Scale bar = 1.0 mm.

Male with small body length: 1.9 ± 0.6 mm (1.4-2.4 mm, n = 10) and wing just longer than body: 2.2 ± 0.5 mm (1.7 -2.5 mm, n = 10) setae covering body and legs is fine and short (Fig. 1). Female unknown. *Head*. Eye dark brown, occiput black, covered with pale yellow pile; ocellar tubercle black; ocelli white, frons dark brown, antennae light brown and placed on mediodorsal eye margin, face dark brown with pale yellow pile, clypeus light brown, bare and shorter than the antennae, mouthparts yellow and strongly reduced. *Thorax*. Uniformly dark brown with covering pale yellow setae; coxae light brown, femora light brown with apex yellow, tibia light brown with basal third yellow , tarsi light brown, basal and apical tarsomeres longer than middle tarsomeres, lower calypter translucent white and covered with pale yellow setae, halter pale yellow. *Wing*. (Fig. 2) All wing veins pale yellow except costa, subcosta and R_1_ pale brown; pterostigma darker. *Abdomen*. Background color brown tergites I-II entirely brown, tergite III with posteriomedial portion yellow, tergites IV-VI medially yellow, sternites brown.

##### Comments.

Based on an examination of the voucher specimens of *Sphaerops* sequenced by Winterton et al. (2007) these were representatives of *Sphaerops micella* sp. n. not *Sphaerops appendiculata* as reported. Therefore the taxon associated with Genbank accession numbers AY140877, AF539875, AY144436, and AY144403 will be changed to *Sphaerops micella* sp. n.

**Etymology.** The species epithet is derived from the Latin: *micella* (diminutive feminine) meaning; little, crumb, or small, in reference to the minute size of this species relative to *Sphaerops appendiculata*.

#### Sphaerops appendiculata

Philippi, 1865

http://species-id.net/wiki/Sphaerops_appendiculata

**Figure 3.**
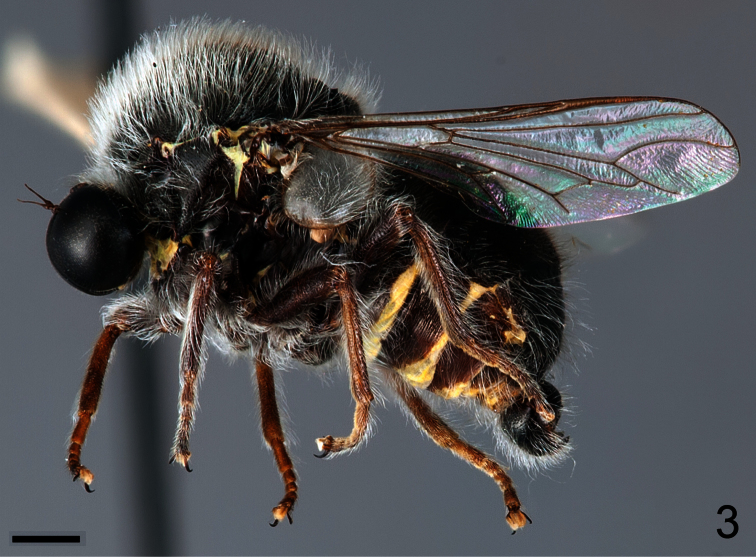
*Sphaerops appendiculata* Philippi, 1865, male, lateral view. Scale bar = 1.0 mm.

Figs 3 [Fig F4] –5 24

##### Material examined.

CHILE; Santiago Prov.; 3 km N. El Arrayan; 7.ix.1966; 1150 m; 33°21'S, 70°28'W; EI Schlinger, ME Irwin. [reared from Sequestriidae (Araneae), remains of spider host pinned with specimen], (EIS # 2951, 2952 (host); 1 ♂, CAS); [Chile] Santiago, 15.xi.[19]39, Stuardo (EIS # 2974; 1 ♂, CAS); [Chile] El Canalo, 15.x.1933; Stuardo (EIS # 2978; 1 ♂, CAS).

##### Diagnosis.

Pile covering body and legs is much longer and denser than in *Sphaerops micella* **sp. n.** (Fig. 3). Antennae are inserted on the head near but not immediately adjacent to the ocellar tubercle. This species is also much larger (6.3 vs 1.9 mm), has brown rather than yellow wing veins, and the wing veins CuA_2_ and A_1_ join near the wing margin.

##### Description.

**Figure 4. F4:**
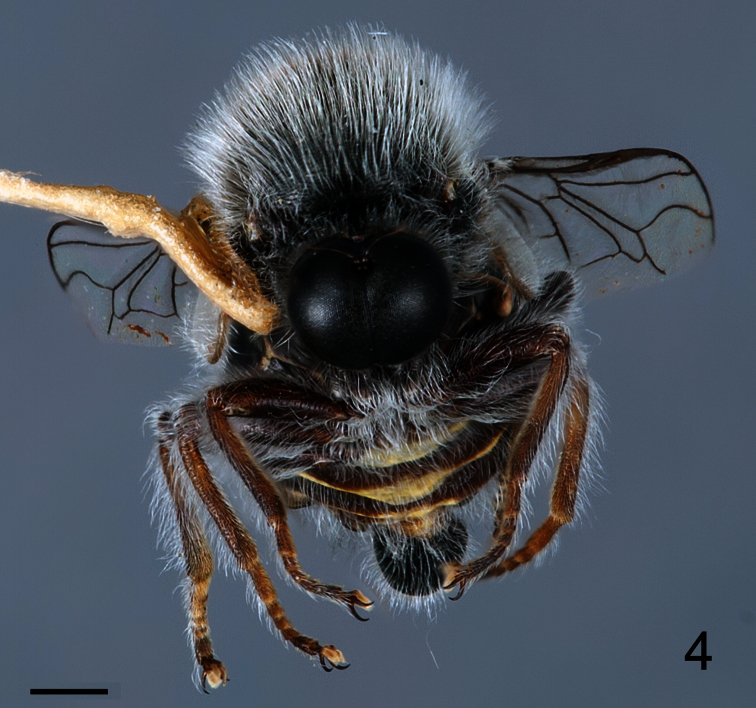
*Sphaerops appendiculata* Philippi, 1865, male, anterior view. Scale bar = 1.0 mm.

**Figure 5.**
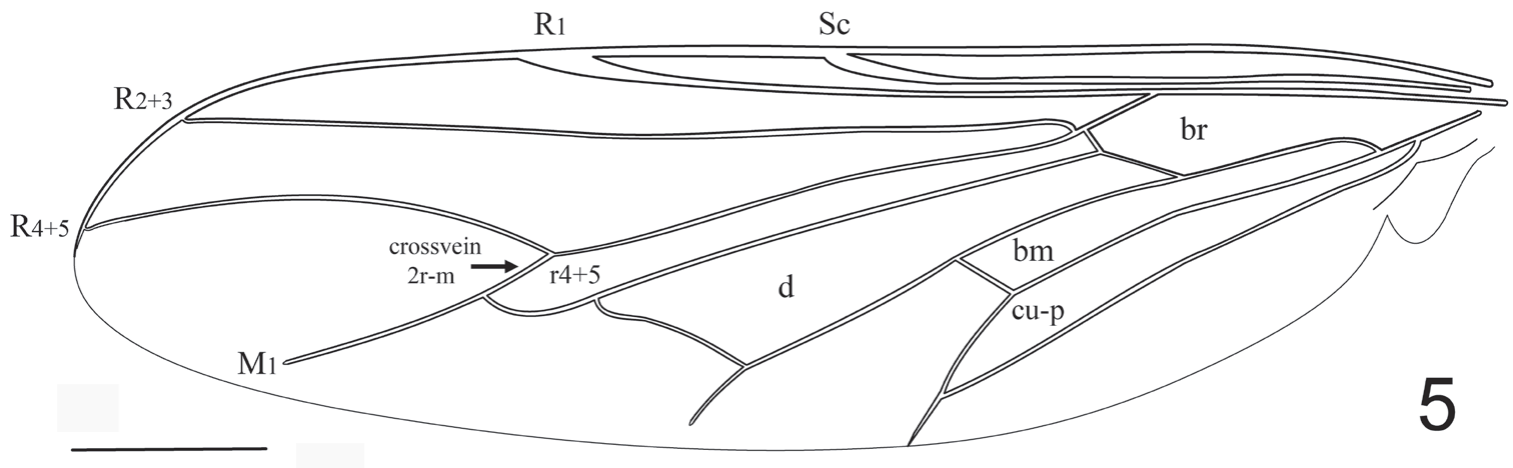
*Sphaerops appendiculata* Philippi, 1865, wing. Scale bar = 1.0 mm.

Male with medium body size: (Fig. 3) 6.3 ± 1.2 mm (5.8 - 7.0 mm, n = 3) and wing shorter than the body: 5.9 ± 1.4 mm (5.1 - 6.4 mm, n = 3). *Head*. (Fig. 4) Eye dark brown, occiput and ocellar tubercle dark brown, covered with pale yellow pile; ocelli light brown, frons dark brown and strongly reduced, antennae light brown, face dark brown with pale yellow pile, clypeus dark brown, bare and as long as scape and pedicel combined, mouthparts yellow and strongly reduced. *Thorax*. Uniformly dark brown with dense covering pale yellow pile; coxae dark brown, femora, tibia and tarsi light brown, basal tarsomere longer than remaining tarsomeres, lower calypter pale yellow with light brown margin and covered with dense pale yellow pile, halter yellow. *Wing*. (Fig. 5) All wing veins brown. *Abdomen*. Tergites dark brown, sternites dark brown with posterior margin yellow.

### Subfamily Philopotinae Schiner, 1868

#### Neophilopota

Schlinger gen. n.

urn:lsid:zoobank.org:act:793E27B2-FC10-4CE5-AAD2-F60E07C81901

http://species-id.net/wiki/Neophilopota

**Figure 6.**
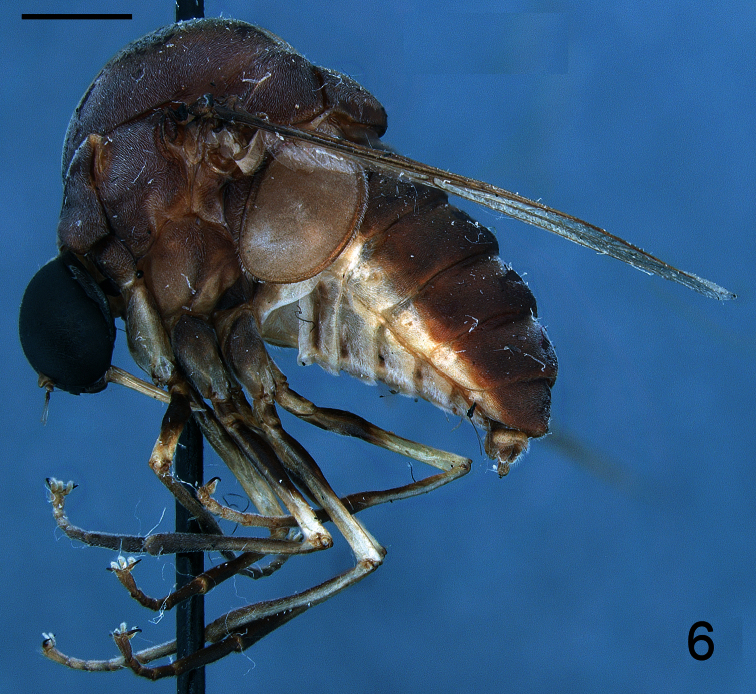
*Neophilopota brevirostris* Schlinger sp. n., male holotype, lateral view. Scale bar = 2.0 mm.

Figs 6 [Fig F7] [Fig F8] [Fig F9] –10

##### Type species.

*Neophilopota brevirostris* sp. n., by present designation.

##### Diagnosis.

*Neophilopota* gen. n.is an endemic Mexican genus similar to *Quasi*, *Oligoneura* and *Philopota*. It can be easily distinguished from the Central American genus *Quasi* as *Neophilopota* has pilose eyes and well developed mouthparts (forming an elongate proboscis). It shows greater similarity to both the Palearctic *Oligoneura* and the Neotropical *Philopota* in having elongate mouthparts and the ocellar tubercle poorly developed. It is easily distinguished from *Philopota* by having the frons as long as wide (though shorter than the antennae), the insertion of the antennae on the lower part of the frons, the lower facial margin wider in the upper portion than in lateral portions, and the clypeus shorter than the antennae. It can be readily differentiated from *Oligoneura* by the absence of the palpi, the presence of pile on the frons, the insertion of antennae on the lower portion of the head, the clypeus being shorter than the antennae and the legs greatly elongated. *Neophilopota* was referred to as ‘New Genus A’ in the *Manual of Central American Diptera* (Schlinger 2009).

##### Description.

**Figure 7. F7:**
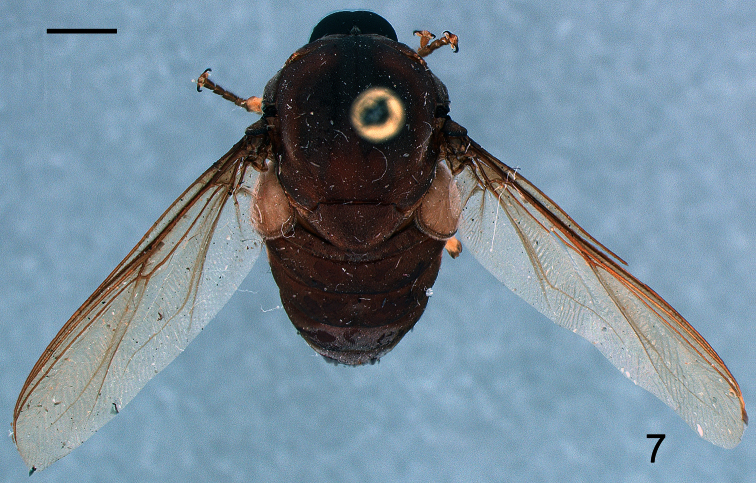
*Neophilopota brevirostris* Schlinger sp. n., male holotype, dorsal view. Scale bar = 2.0 mm.

**Figure 8. F8:**
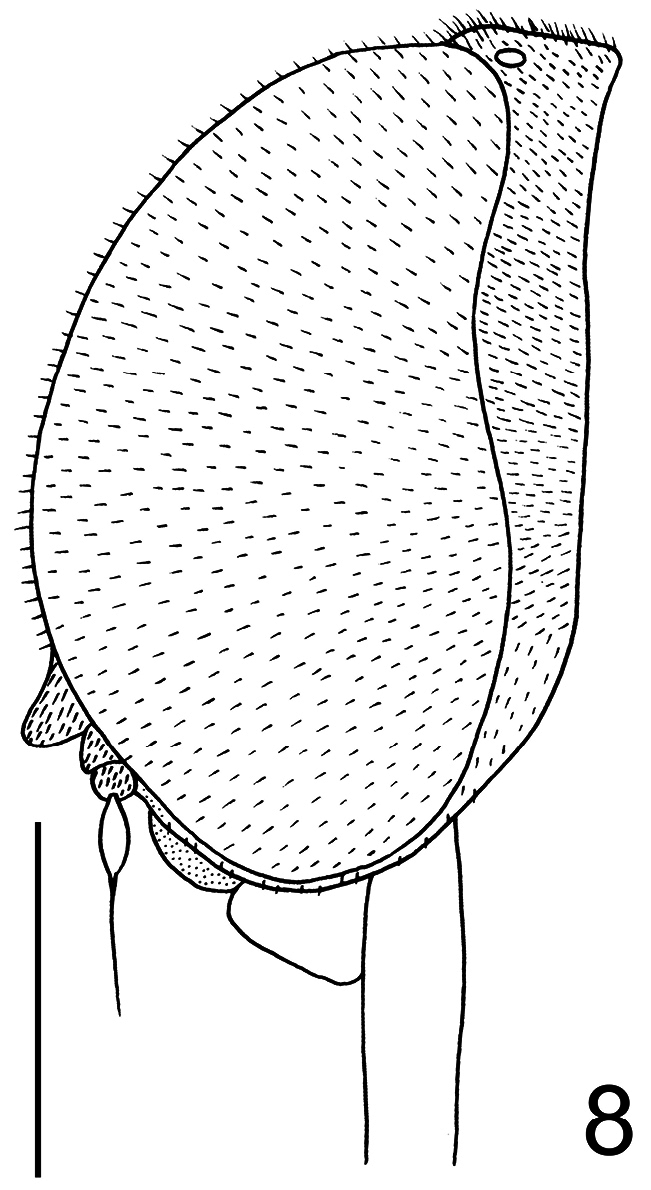
*Neophilopota brevirostris* Schlinger sp. n., head, lateral view. Scale bar = 1.0 mm.

**Figure 9. F9:**
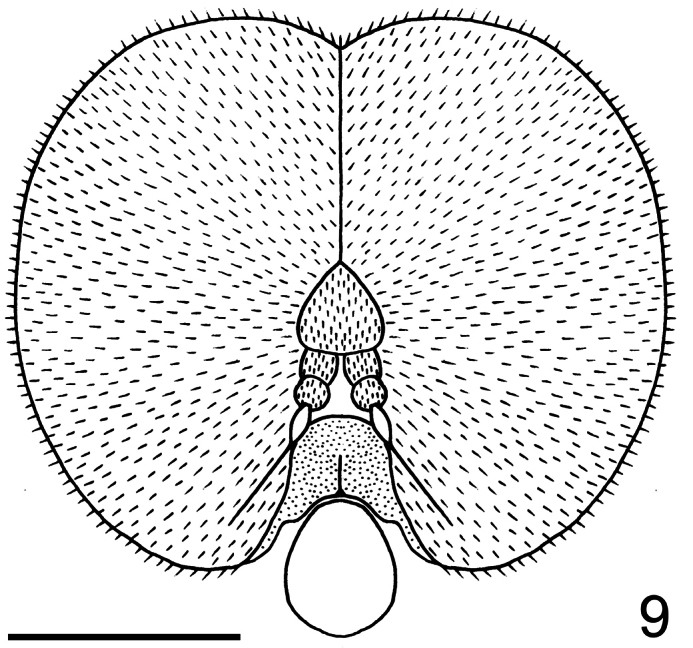
*Neophilopota brevirostris* Schlinger sp. n., head, frontal view. Scale bar = 1.0 mm.

**Figure 10.**
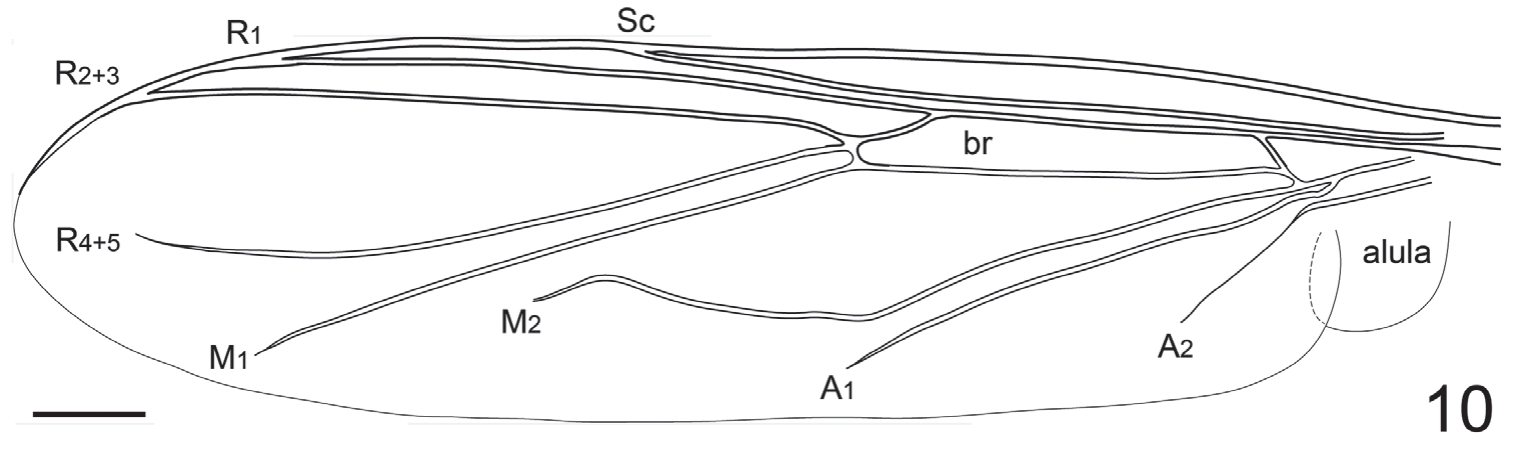
*Neophilopota brevirostris* Schlinger sp. n., wing, dorsal view. Scale bar = 1.0 mm.

Body shape arched (Fig. 6); coloration non-metallic. Head width slightly smaller than thorax width (Fig. 7); nearly spherical; ocellar tubercle slightly raised, rounded with three ocelli (Fig. 8); postocular ridge and occiput extended posteriorly into acute ridge; posterior margin of eye rounded; eye sparsely pilose; eyes contiguous above antennal base; not contiguous below; palpus absent; proboscis length subequal to or slightly greater than head length; without pile, or setae barely evident; antennae located nearer to mouthparts (Figs 8, 9); flagellum stylate; apex lacking terminal setae; scapes not fused together; postpronotal lobes enlarged, medially contiguous forming a collar; antepronotum narrow; subscutellum enlarged; legs greatly elongated; tibial spines absent; pulvilli present; wing markings and microtrichia absent (Fig. 10); costa ending near wing apex; costal margin straight; humeral crossvein present; radial veins straight, R_1_ not inflated distally; veins R_4_ and R_5_ present as single vein; crossvein 2r-m absent; two M veins present, not reaching wing margin; discal cell open distally; cell m_3_ absent; CuA_1_ reduced, not reaching wing margin; CuA_2_ reduced; anal lobe well developed; alula well developed; abdomen conical, tapering towards apex; tergites smooth, rounded.

##### Included species.

*Neophilopota brevirostris* sp. n.

##### Etymology.

The prefix of the genus epithet (*neo*) is derived from the Latin for “new”, referring to this being a new genus. The suffix, -*philopota*, is used in reference to *Neophilopota*’s similarity to *Philopota*.

#### Neophilopota brevirostris

Schlinger sp. n.

urn:lsid:zoobank.org:act:4305FC9E-7691-433D-9737-40F322F8C2CB

http://species-id.net/wiki/Neophilopota_brevirostris

Figs 6 [Fig F7] [Fig F8] [Fig F9] –10 24

##### Material examined.

**Holotype** male: Top label “Fortin de las / Flores, Ver., / Mex.”. “Doyen and / Foster Collec.”. “USNM”. Middle label bright green “Acroceridae / E.I. Schlinger / Specimen / 004294”. Bottom label: red “HOLOTYPE ♂ / Neophilopota/ brevirostris/ Schlinger” (USNM).

**Paratype** male: “Rio Metlac, MEX. / Fortin de las Flores / Veracruz / VIII-17-1965 / L.R. Gillogly”. “E.I. Schlinger / Collection”. red label “Genitalia / Dissection No. 78-6-22k. / by E.I. Schlinger”. bright green label “Acroceridae / E.I. Schlinger / Specimen / 004293” . Bottom label: yellow “Paratype ♂ / Neophilopota / brevirostris / Schlinger” (CAS). Genitalia dissected and placed in glycerin in glass microvial on pin with specimen.

##### Description.

Male with medium body size (male body: 9.5–12.3 (holotype) mm; n = 2) and wing longer than the body (male wing: 10.8–15.1 (holotype) mm; n = 2). *Head*. (Figs 8, 9) Ocellar tubercle brown; antennae brown (Fig. 6), longer than frons; postocular ridge brown, wider than clypeus; face black; clypeus brown, shorter than antennae and bare. *Thorax*. (Fig. 7) Brown with dark brown markings; legs elongate; coxae brown; femora brown with apex light brown; tibia brown; tarsi brown; lower calypter brown with dark brown margin. *Wing*. (Fig. 10) Infuscate, without markings; wing veins brown. *Abdomen*. Tergite I entirely brown; tergites II-VI brown with lateral margin yellow; sternites yellow.

##### Comments.

The proboscis in the holotype is broken (Fig. 6), but in the paratype it is longer than the head height and shorter than the body length.

##### Etymology.

The species epithet is derived from the Latin: *brevis* (short) and *rostris* (beak), in reference to the short length of proboscis in comparison to species of *Philopota*.

### Subfamily Panopinae Schiner, 1868

#### Coquena

Schlinger gen. n.

urn:lsid:zoobank.org:act:B51436F7-465D-498C-B56A-2074E2986AF8

http://species-id.net/wiki/Coquena

**Figure 11.**
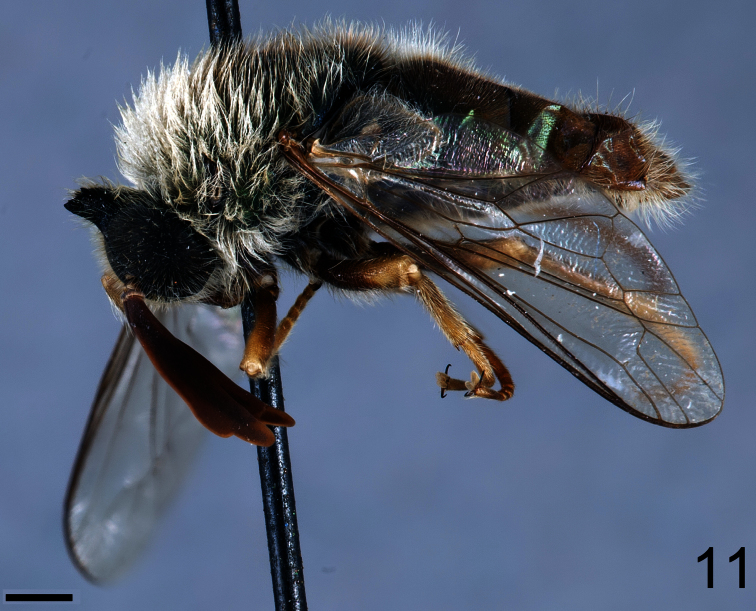
*Coquena stangei* Schlinger sp. n., male holotype, lateral view. Scale bar = 1.0 mm.

Figs 11 [Fig F12] [Fig F13] [Fig F14] –15

##### Type species.

*Coquena stangei* Schlinger sp. n.

##### Diagnosis.

*Coquena* is a South American genus readily distinguished from other panopine genera by the minute mouthparts, the ocellar tubercle extremely raised and the iridescent body color. It is closely related to the Neotropical genera *Lasioides* and *Pteropexus*,the Nearctic genus *Eulonchus*, and the New World genus *Lasia*, as it shares the same wing venation. However it does not have the elongate proboscis present in these genera. *Coquena* and *Lasioides* are closely related, monotypic, genera. The mouthparts in *Coquena stangei* are strongly reduced, whereas they are elongate in *Lasioides peruanus* Gil Collado, 1928. *Coquena* shares some attributes with *Lasia* such as the presence of an alula and having the eyes separate below the antennae. It also shares several characteristics with *Eulonchus*, principally the extremely raised ocellar tubercle and presence of maxillary palpi.

##### Description.

**Figure 12. F12:**
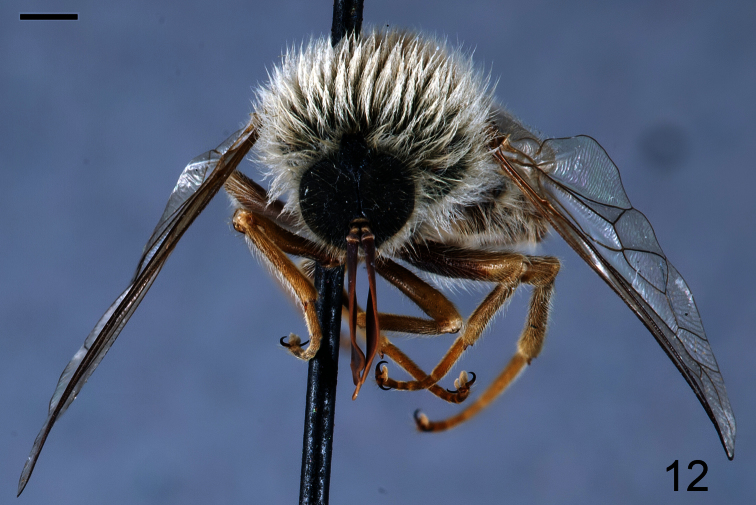
*Coquena stangei* Schlinger sp. n., male holotype, anterior view. Scale bar = 1.0 mm.

**Figure 13. F13:**
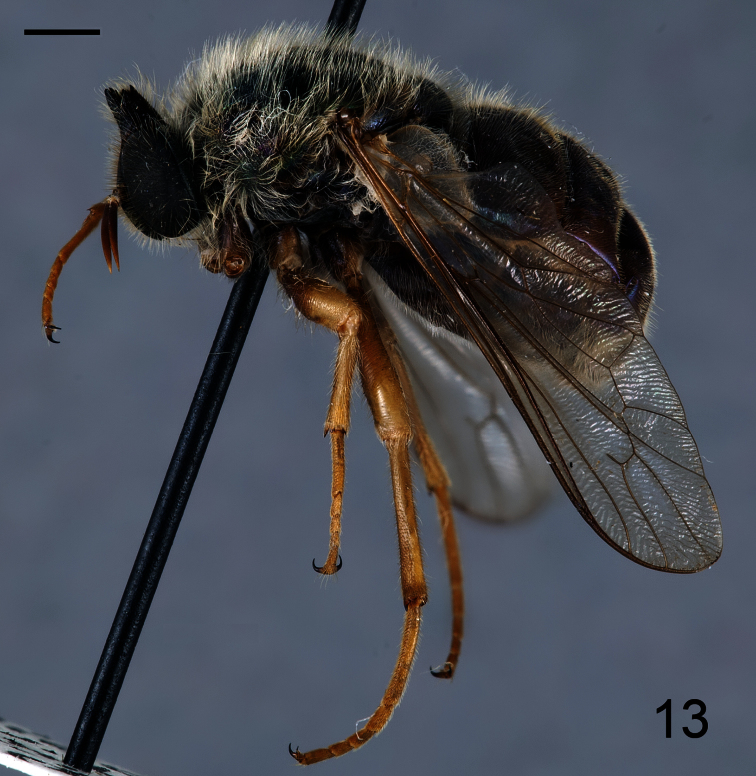
*Coquena stangei* Schlinger sp. n., female paratype, lateral view. Scale bar = 1.0 mm.

**Figure 14. F14:**
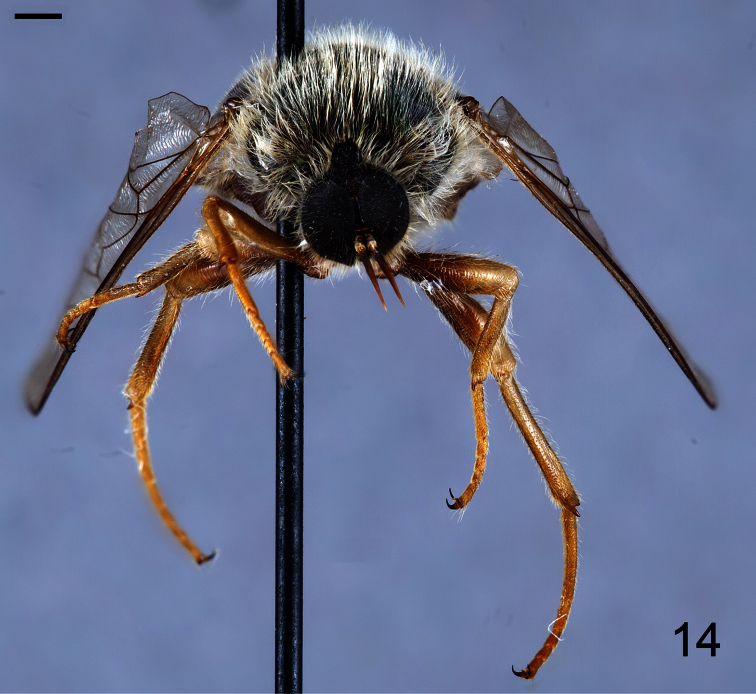
*Coquena stangei* Schlinger sp. n., female paratype, anterior view. Scale bar = 1.0 mm.

**Figure 15.**
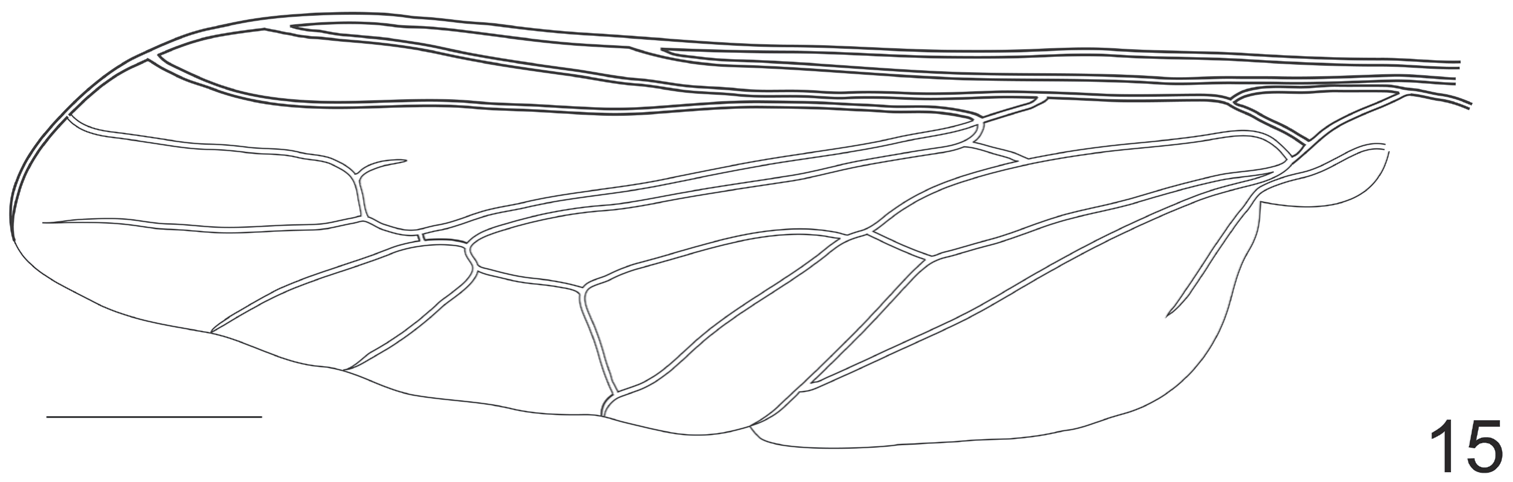
*Coquena stangei* Schlinger sp. n., wing. Scale bar = 1.0 mm.

Body shape not arched (Figs 11, 13); coloration metallic iridescent. Head width slightly narrower than thorax width (Figs 12, 14); hemispherical; ocellar tubercle shape greatly raised and irregularly shaped, 1/4 to 1/2 as high as head; two ocelli, anterior ocellus absent; postocular ridge and occiput rounded; posterior margin of eye rounded; eye densely pilose; eyes not contiguous above antennal base; palpus present; proboscis greatly reduced, without pile, or setae barely evident; antennae located on middle of frons; flagellum elongate, paddle-shaped, much larger in male, apex lacking terminal setae; scapes not fused together; postpronotal lobes not enlarged or contiguous medially; antepronotum narrow; subscutellum barely visible beneath scutellum; legs not elongate; tibial spines present apically; pulvilli present; wing markings and microtrichia absent (Fig. 15); costa circumscribing entire wing margin, costal margin straight; humeral crossvein present; radial veins straight; R_1_ not inflated distally; veins R_4_ and R_5_ present; crossvein 2r-m present between M_1_ and R_4+5_, bisecting cell r_4+5_; cell formed by 2r-m present, narrow and elongate; M_1_, M_2_ and M_3_ present (M_3_ fused with CuA_1_), reaching wing margin; discal cell closed completely; cell m_3_ present; CuA_1_ joining M_3_, and running to margin; CuA_2_ fused to A_1_ before wing margin and then running to margin; anal lobe well developed; alula well developed; abdomen greatly rounded, inflated, tergites smooth.

##### Etymology.

The genus epithet is derived from the *Coquena* legend of north-western Argentina. *Coquena* was the son of Mother Earth and was portrayed as a short man dressed in a hat and bright colored poncho. The iridescent coloration, hat-like ocellar tubercle and type locality in northwest Argentina of the type species led to the choice of this name for the genus.

#### Coquena stangei

Schlinger sp. n.

urn:lsid:zoobank.org:act:51AC7C00-FAF5-4581-87B1-7D893266698F

http://species-id.net/wiki/Coquena_stangei

Figs 11 [Fig F12] [Fig F13] [Fig F14] –15 24

##### Material examined.

**Holotype** male: Top label: “*El Solidad, Argentina/ 11 km. W Las Cejas/ Tucuman Prov./ IV-30 to V-13-1967/ L. Stange (malaise)”* [26.895318°S, 64.835332°W]. Second label: “Genitalia *IX-10-69*/ Dissection No. *9*/ by E.I. Schlinger”. Third label: bright green “ACROCERIDAE/ E.I. Schlinger/ Specimen #/ 013435”. Bottom label: red “HOLOTYPE ♂/ *Coquena stangei*/ Schlinger new species/ Det. E.I. Schlinger 2012” (CAS). Genitalia dissected and placed in glycerin in glass microvial on pin with specimen.

##### Paratypes.

Four females, same data as holotype except: genitalia not dissected; EIS specimen numbers are: 013436, 013437, 013438, 013441; and with yellow paratype labels.

##### Description.

Male holotype with medium body size (Fig. 11): 7.80 mm and wing shorter than body: 7.0 mm. Female with medium body size (Fig. 13): 6.7 ± 1.21 mm (6.0 - 7.5 mm, n= 4) and wing longer than the body: 7.8 ± 1.2 mm (7.0 - 8.5 mm, n= 4). *Head*. Eyes dark brown and densely covered with pale yellow pile (Figs 12, 14), occiput and ocellar tubercle dark brown and covered with yellow pile, ocelli light brown, frons dark brown with region adjacent to antennae yellow, scape and pedicel light brown with apex yellow, pedicel with light brown pile, flagellum light brown, male flagellum length ~2× height of the eye and petal-shaped, female flagellum length ½ the height of the eye and tapering to apex. Face dark brown with yellow pile, clypeus brown, bare and slightly longer than scape and pedicel combined, mouthparts pale yellow and strongly reduced. *Thorax*. Iridescent green and densely covered with long yellow pile. Coxae light brown, femora light brown with apical third yellow, tibia and tarsi light brown. Lower calypter transparent covered with dense yellow pile, halter yellow. *Wing*. Transparent (clear or pale brown), veins brown (Fig. 13). *Abdomen*. Tergites iridescent brown densely covered with yellow pile; sternites brown and densely covered with yellow pile.

##### Etymology.

This species is named in honor of Dr. Lionel A. Stange, Florida State Collection of Arthropods, who collected the type series.

#### Pialea

Erichson, 1840

http://species-id.net/wiki/Pialea

##### Diagnosis.

Body shape not arched; coloration non-metallic. Head width much narrower than thorax width; hemispherical; ocellar tubercle shape raised, rounded, two ocelli present, anterior ocellus absent; postocular ridge and occiput rounded; posterior margin of eye rounded; eyes densely pilose; not contiguous above antennal base, rarely contiguous below; palpus absent; proboscis length greatly reduced, with sparse pile; antennae located on middle of frons, either nearer to ocellar tubercle or to mouthparts; flagellum elongate, slightly tapered or paddle-shaped; apex with terminal setae present or absent; scapes fused; postpronotal lobes not enlarged or contiguous medially; antepronotum narrow; subscutellum barely visible beneath scutellum; legs not elongated; tibial spines present apically; pulvilli present; wing markings and microtrichia absent; costal vein ending near wing apex; costal margin straight; humeral crossvein absent; radial veins straight; R_1_ not inflated distally; R_4+5_ originating at apex of basal cell r_4+5_ and then forking into veins R_4_ and R_5_ (Fig. 36); crossvein 2r-m present between M_1_ and R_4+5_, bisecting cell r_4+5_; cell formed by 2r-m narrow, elongate; R_4_ without spur vein; M_1_, M_2_ and M_3_ present (M_3_ fused with CuA_1_), rarely one M vein or two M veins present, all typically reaching wing margin; discal cell closed; cell m_3_ present, CuA_1_ joining M_3_ and running towards margin; CuA_2_ fused to A_1_ before wing margin and running towards margin; anal lobe well developed; alula weakly developed; abdomen greatly rounded, inflated; tergites smooth, rounded.

##### Comments.

*Pialea* is a relatively rare South American genus that comprises four species described from Brazil and another one described from Ecuador. The genus was described by Erichson (1840) for his new species *Pialea lomata* from Brazil. Later, Westwood (1876) named a second species, *Pialea lutescens*, also from Brazil. Two more species from the Oriental Region were later described in this genus, *Pialea jardinei* and *Pialea auripila* (Brunetti 1912), but they were subsequently transferred to *Astomella* Latreille, 1809and *Astomelloides* Schlinger, 1959, respectively (Schlinger 1956, 1959). Schlinger (1956) revised the genus and described three more species, *Pialea antiqua*, *Pialea capitella* and *Pialea ecuadoriensis*. *Pialea* is presumably closely related to *Stenopialea* Speiser, 1920 (a South African endemic genus) and *Archipialea* Schlinger, 1973 (Chile) based on antennal and wing characters (Schlinger 1973; Barraclough 1985). *Pialea* are characterized by the fusion of the scapes and the dichoptic eyes (except in *Pialea capitella*). Species in this genus also show strong sexual dimorphism in the length, insertion and shape of the antennae, the length of the abdomen and the color and maculation of the body.

##### Key to species of *Pialea*

**Table:** 

1	M_1_ present	2
–	M_1_ absent	4
2. (1)	Tibia and first tarsomere of hind leg greatly swollen (Figs 20, 22), twice as wide as the second tarsomere; additional r-m crossvein (2r-m) present (Fig. 23) (W. Venezuela)	*Pialea corbiculata* Schlinger, sp. n.
–	Tibia and first tarsomere of hind leg not swollen (Figs 16, 35), almost as wide as the second tarsomere; additional r-m crossvein (2r-m) absent (Figs 19, 28)	3
3. (2)	Ocellar tubercle raised; thorax yellow with two longitudinal black stripes (Brazil)	*Pialea lutescens* Westwood, 1876
–	Ocellar tubercle not raised; thorax brown, without stripes (Figs 31–34) (Brazil)	*Pialea antiqua* Schlinger, 1956
4. (1)	R_2+3_ complete, reaching wing margin (Fig. 36) (Brazil)	*Pialea lomata* Erichson, 1840
–	R_2+3_ incomplete, not reaching wing margin (Fig. 19)	5
5. (4)	Eyes holoptic below antennae (Brazil)	*Pialea capitella* Schlinger, 1956
–	Eyes separated below antennae	6
6. (5)	M_2_ absent; antennae inserted on the top of the head in male (i.e., Fig. 34) or middle of head in female (i.e. Fig. 33) (Ecuador)	*Pialea ecuadorensis* Schlinger, 1956
–	M_2_ present (Fig. 19); antennae inserted in the middle of the head (only known from female) (Fig. 18) (W. Venezuela)	*Pialea brunea* Schlinger, sp. n.

#### Pialea brunea

Schlinger sp. n.

urn:lsid:zoobank.org:act:992C5682-3628-4BAC-9E2A-1DD703AD80F7

http://species-id.net/wiki/Pialea_brunea

**Figure 16.**
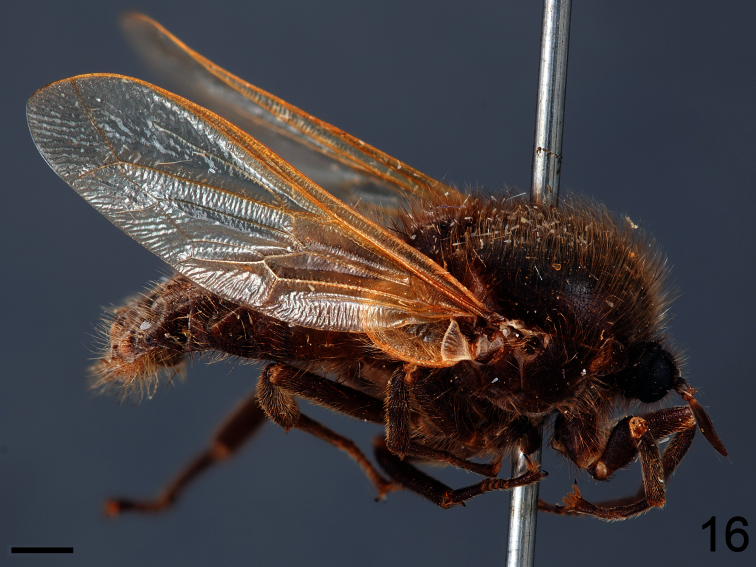
*Pialea brunea* Schlinger sp. n., female holotype, lateral view. Scale bar = 1.0 mm.

Figs 16 [Fig F17] [Fig F18] –19 24

##### Material examined.

**Holotype** female: Top label: “*Venezuela* / *Páramo* / *La Negra* / *Lichy col*. / [vertically on left side] *viii.48*”. Bottom label: red “HOLOTYPE ♀/ *Pialea brunea*/ Schlinger new species/ Det. E.I. Schlinger 2012” (CAS).

##### Diagnosis.

**Figure 17. F17:**
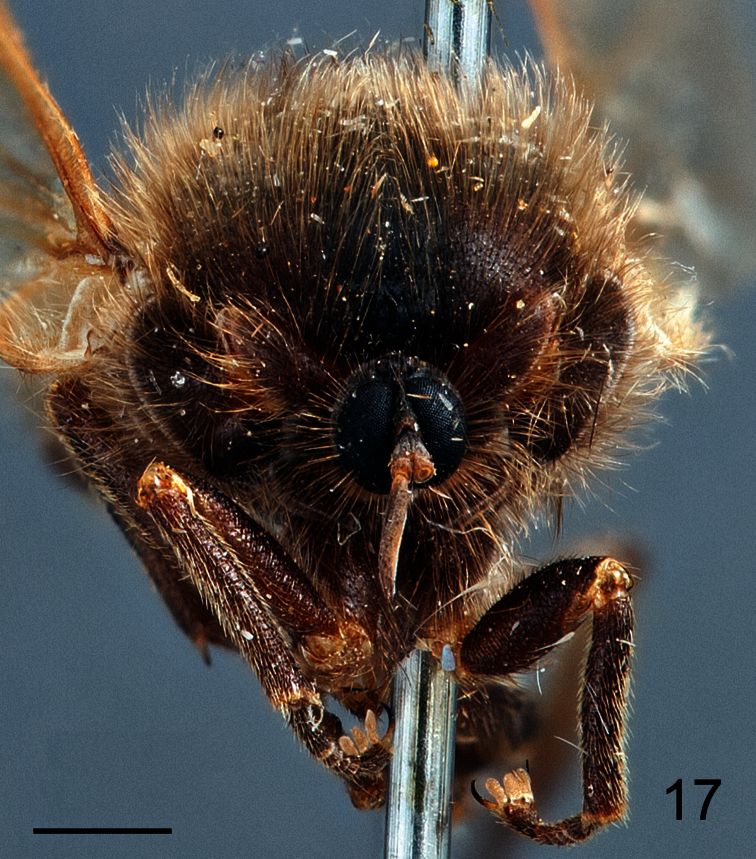
*Pialea brunea* Schlinger sp. n., female holotype, anterior view. Scale bar = 1.0 mm.

**Figure 18. F18:**
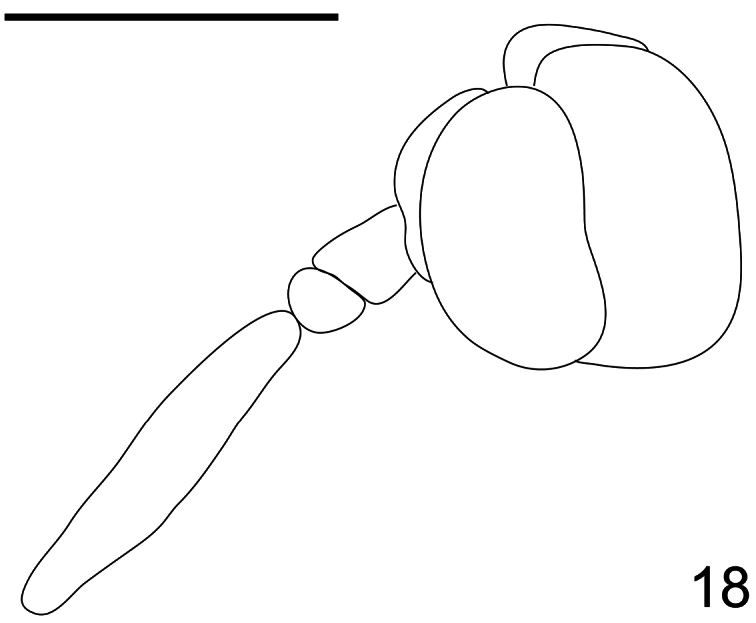
*Pialea brunea* Schlinger sp. n., female, head, lateral view. Scale bar = 1.0 mm.

**Figure 19.**
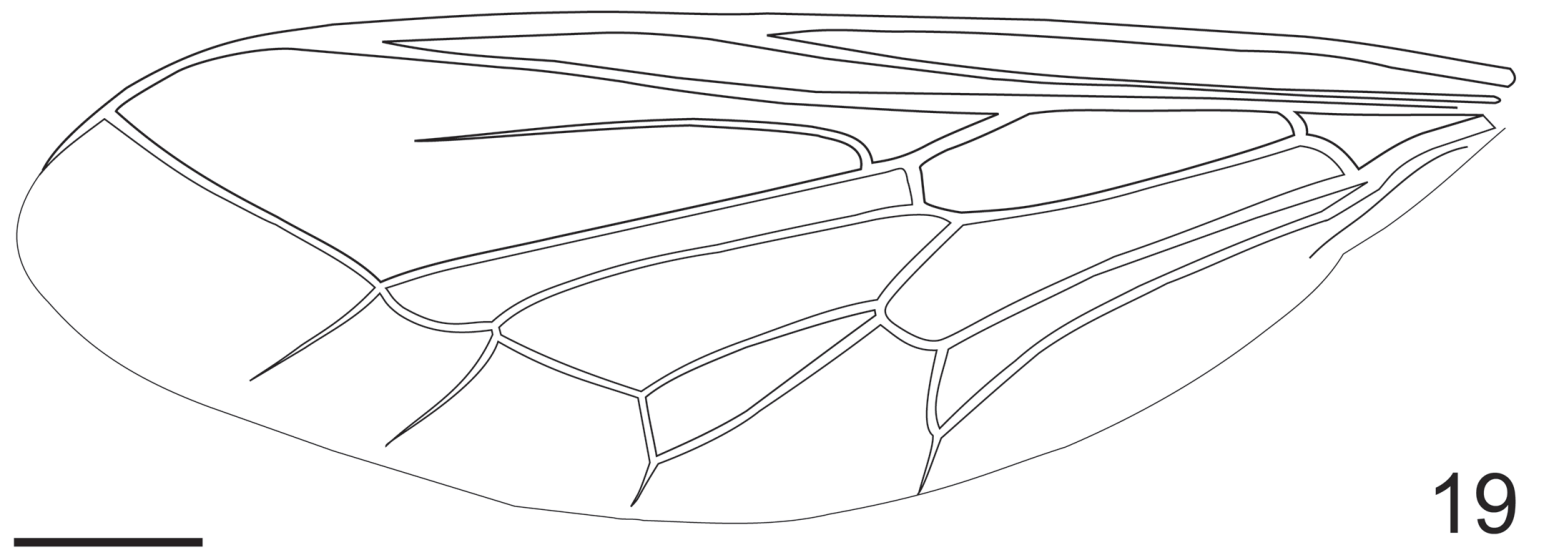
*Pialea brunea* Schlinger sp. n., wing. Scale bar = 1.0 mm.

Antennae inserted in the middle of the head (female, Fig. 17); post pedicel longer than head height (Fig. 18); body entirely brown, without yellow markings; R_2+3_ not reaching wing margin (Fig. 19); M_2_ present; first tarsomere of hind leg much longer than the remaining tarsomeres combined.

##### Description.

Female holotype with medium body size (Fig. 16, female body: 9.2 mm) and wing shorter than body (female wing: 8.2 mm). *Head*. Eyes black and densely covered with long (equal to length of scape) brown pile (Fig. 17), occiput and ocellar tubercle dark brown and covered with brown pile, ocelli brown, frons brown, scape and pedicel brown, pedicel with several long setae, flagellum brown and tapering to apex which bears setae (Fig. 18). Face dark brown with brown pile; clypeus dark brown, half the length of the scape and covered with fine setae; mouthparts light brown and strongly reduced. *Thorax*. Uniformly light brown and densely covered with long brown pile. Legs brown and densely covered with long brown pile. Lower calypter brown and densely covered with yellow pile; halter light brown. *Wing*. Transparent with light brown wing veins (Fig. 19). R_2+3_ incomplete, not reaching wing margin, M_1_ absent, M_2_ present. *Abdomen*. Both tergites and sternites uniformly dark brown.

##### Comments.

*Pialea brunea* is closely related to *Pialea capitella* Schlinger, 1956 and *Pialea ecuadorensis* Schlinger, 1956, sharing with these species the absence of wing vein M_1_. It differs from *Pialea capitella* in the eyes being separate below the antennae, and from *Pialea ecuadorensis* in the overall brown coloration and presence of M_2_.

##### Etymology.

The specific epithet is derived from the Latin, *bruneus* – brown; referring to the distinctive entirely brown coloration of the body, which lacks yellow markings.

#### Pialea corbiculata

Schlinger sp. n.

urn:lsid:zoobank.org:act:9188FD90-4D9A-430B-A014-BE5D13D604CE

http://species-id.net/wiki/Pialea_corbiculata

**Figure 20.**
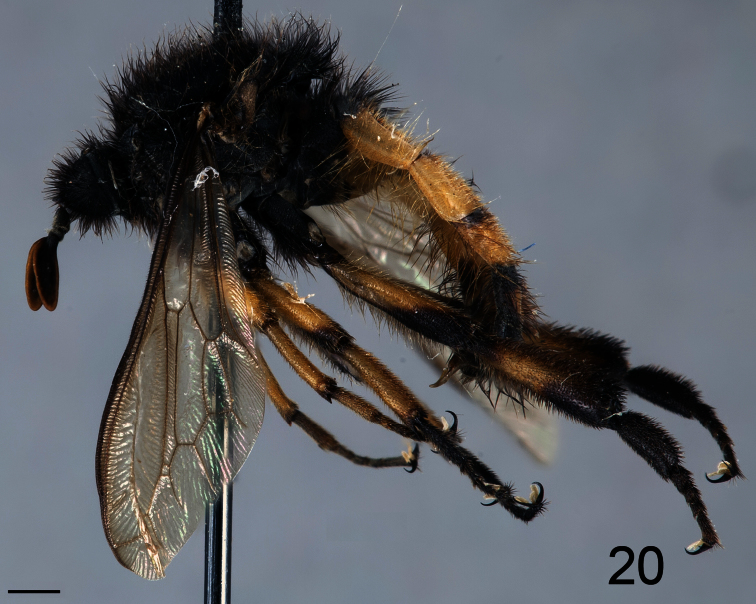
*Pialea corbiculata* Schlinger sp. n., male holotype, lateral view. Scale bar = 1.0 mm.

Figs 20 [Fig F21] [Fig F22] [Fig F23] –24

##### Material examined.

**Holotype** male: Top label: “*VENEZUELA* / *Mérida, 3500 m*. / *Páramo Mucubaji* / *nr. Laguna Negra*”. Second label: “*29.iv-3.v.81* / *Malaise trap* / *L. Masner* / *8115*”. Third label: bright green “ACROCERIDAE/ E.I. Schlinger/ Specimen #/ 004215”. Bottom label: red “HOLOTYPE ♂/ *Pialea corbiculata*/ Schlinger new species/ Det. E.I. Schlinger 2012” (CAS).

##### Diagnosis.

Antennae inserted in the middle of the head (male); post pedicel as long as the head height; head, thorax and scutellum black; legs and abdomen black with yellow markings; hind leg with tibia and first tarsomere swollen (Figs 20, 22), twice as wide as the second tarsomere; additional r-m crossvein (2r-m) present.

##### Description.

**Figure 21. F21:**
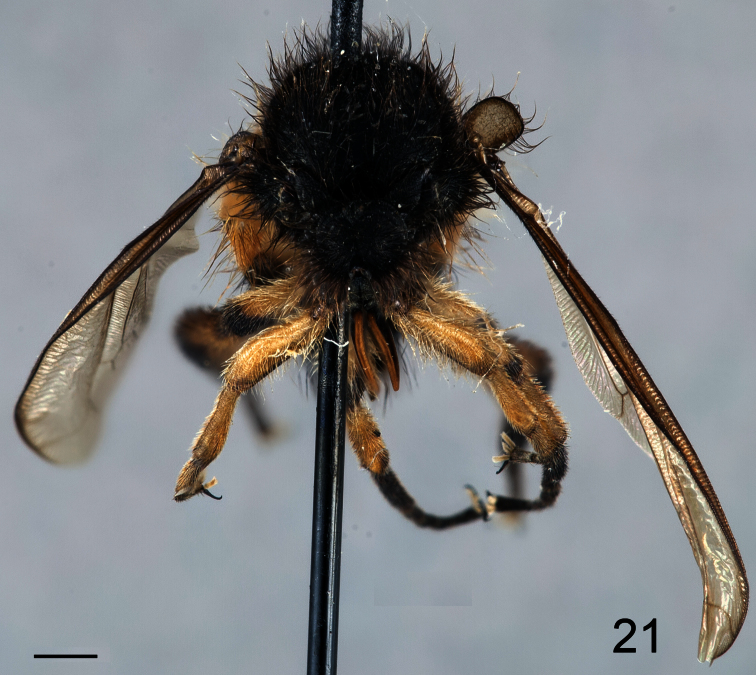
*Pialea corbiculata* Schlinger sp. n., male holotype, anterior view. Scale bar = 1.0 mm.

**Figure 22. F22:**
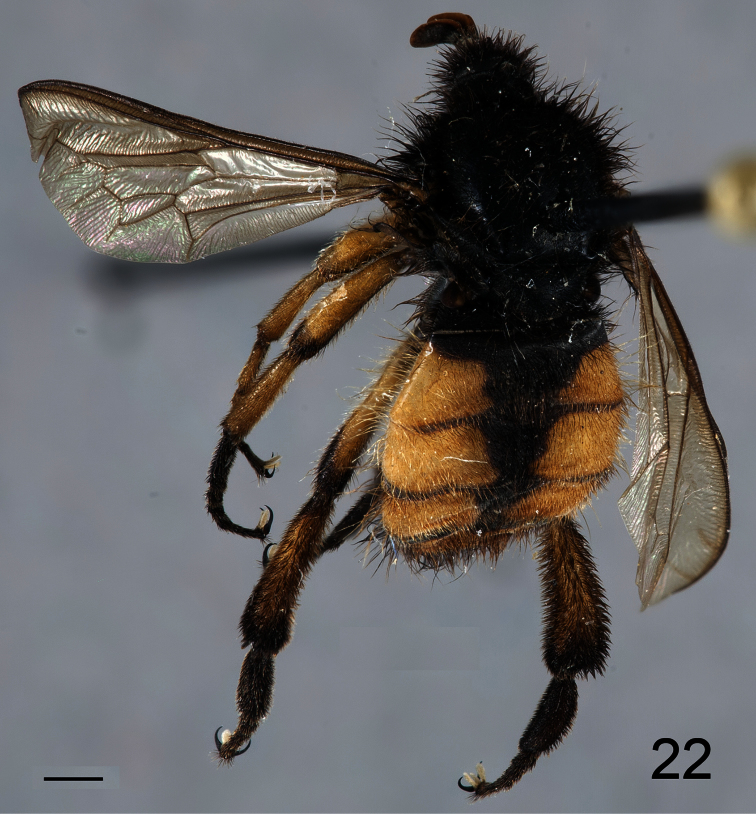
*Pialea corbiculata* Schlinger sp. n., male holotype, dorsal view. Scale bar = 1.0 mm.

Male holotype with medium body size (Fig. 20, male body length: 7.3 mm) and wing longer than body (male wing: 8.6 mm). *Head*. Eyes black and densely covered with dark brown, long (equal to scape length) pile (Fig. 21), occiput and ocellar tubercle dark brown and densely covered with long dark brown pile, ocelli light brown, frons dark brown, scape and pedicel dark brown, pedicel with yellow setae, flagellum light brown, lacking apical setae. Male flagellum petal like, length ~2× length of scape and pedicel combined. Face dark brown with brown pile, clypeus dark brown, with light brown pile and shorter than scape, mouthparts dark brown and strongly reduced. *Thorax*. Dark brown and densely covered with long brown pile (Fig. 22). Coxae dark brown, femora yellow with apex of ventral surface brown, tibia yellow with apex brown, tarsi brown, hind leg longer than fore- and mid-leg and with tibia and first tarsomere swollen (twice as wide as mid-leg). Lower calypter brown and densely covered long brown pile, halter brown. *Wing*. Transparent light brown with brown wing veins; M_1_ and M_2_ present. *Abdomen*. Densely covered with light brown pile (Fig. 22). Tergite I and VI brown, tergites II-V yellow with anterior margin and medial line brown; sternites I and VI brown sternite II–V yellow.

##### Comments.

*Pialea corbiculata* sp. n. is closely related to *Pialea lutescens* Westwood, 1876 and *Pialea antiqua* Schlinger, 1956, based on the presence of wing vein M_1_. It differs from those two species by the swollen tibia and first tarsomere of the hind leg and the presence of an additional r-m crossvein (2r-m), which are unique features within *Pialea*.

##### Etymology.

The species epithet, *corbiculata*, is in reference to the swollen hind tibia and tarsi which resemble the pollen collecting corbicula of many bees.

**Figure 23. F23:**
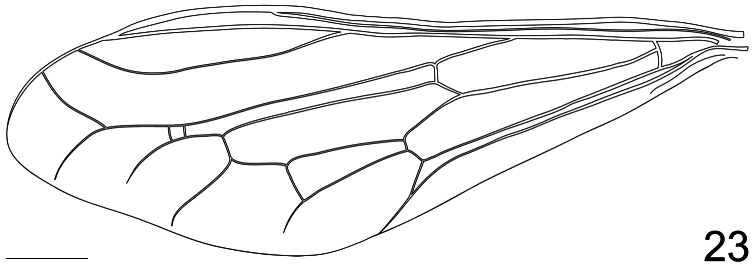
*Pialea corbiculata* Schlinger sp. n., wing. Scale bar = 1.0 mm.

**Figure 24.**
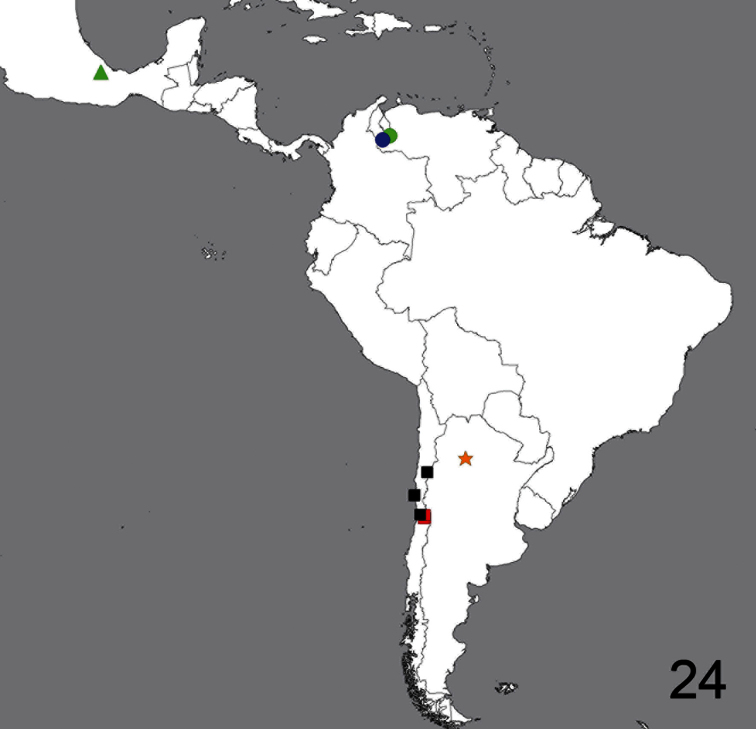
Distribution of *Sphaerops micella* Schlinger sp. n.(black squares), *Sphaerops appendiculata* Philippi, 1865. (red squares), *Neophilopota brevirostris* Schlinger sp. n. (green triangle), *Coquena stangei* Schlinger sp. n.(orange star), *Pialea brunea* Schlinger sp. n. (blue circle) and *Pialea corbiculata* Schlinger sp. n. (green circle).

**Figure 25.**
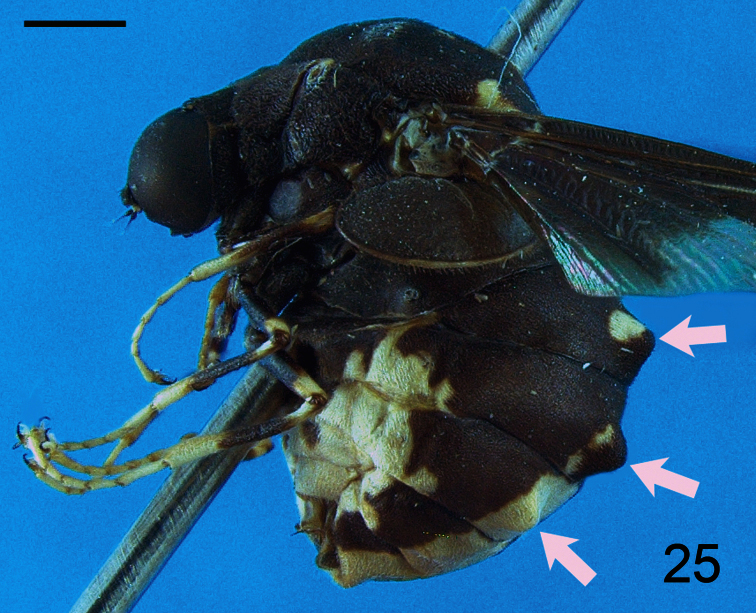
*Terphis nodosa* Erichson, 1840, female, lateral view. Scale bar = 1.0 mm.

**Figure 26.**
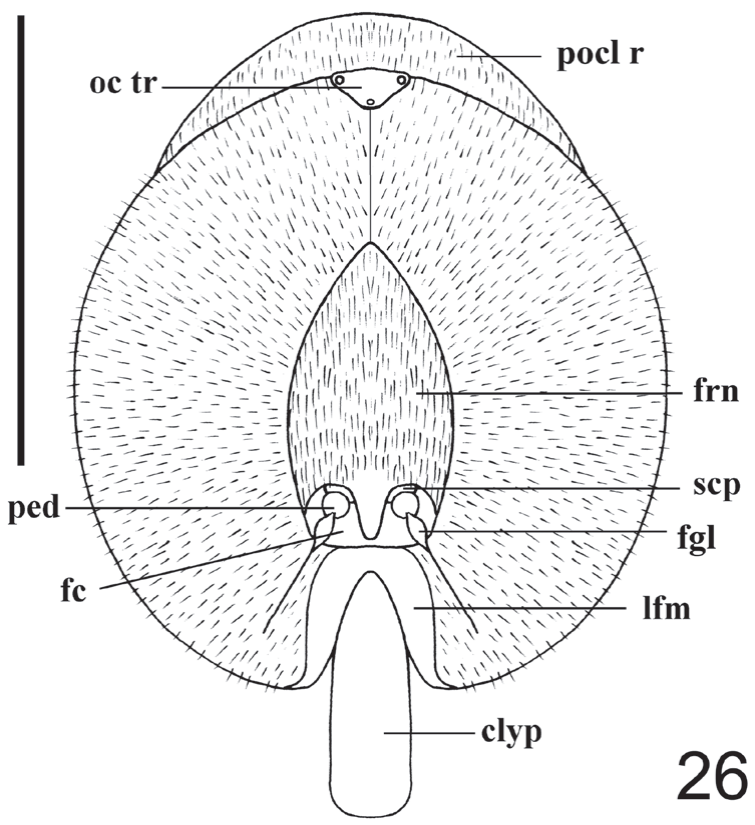
*Philopota conica* Wiedemann, 1830, female, head, anterior view. Scale bar = 1.0 mm. **clyp** clypeus **fc** face **flg** flagellum **frn** frons **lfm** lower facial margin **oc tr** ocellar triangle **ped** pedicel **pocl r** postocular ridge **scp** scape.

**Figure 27.**
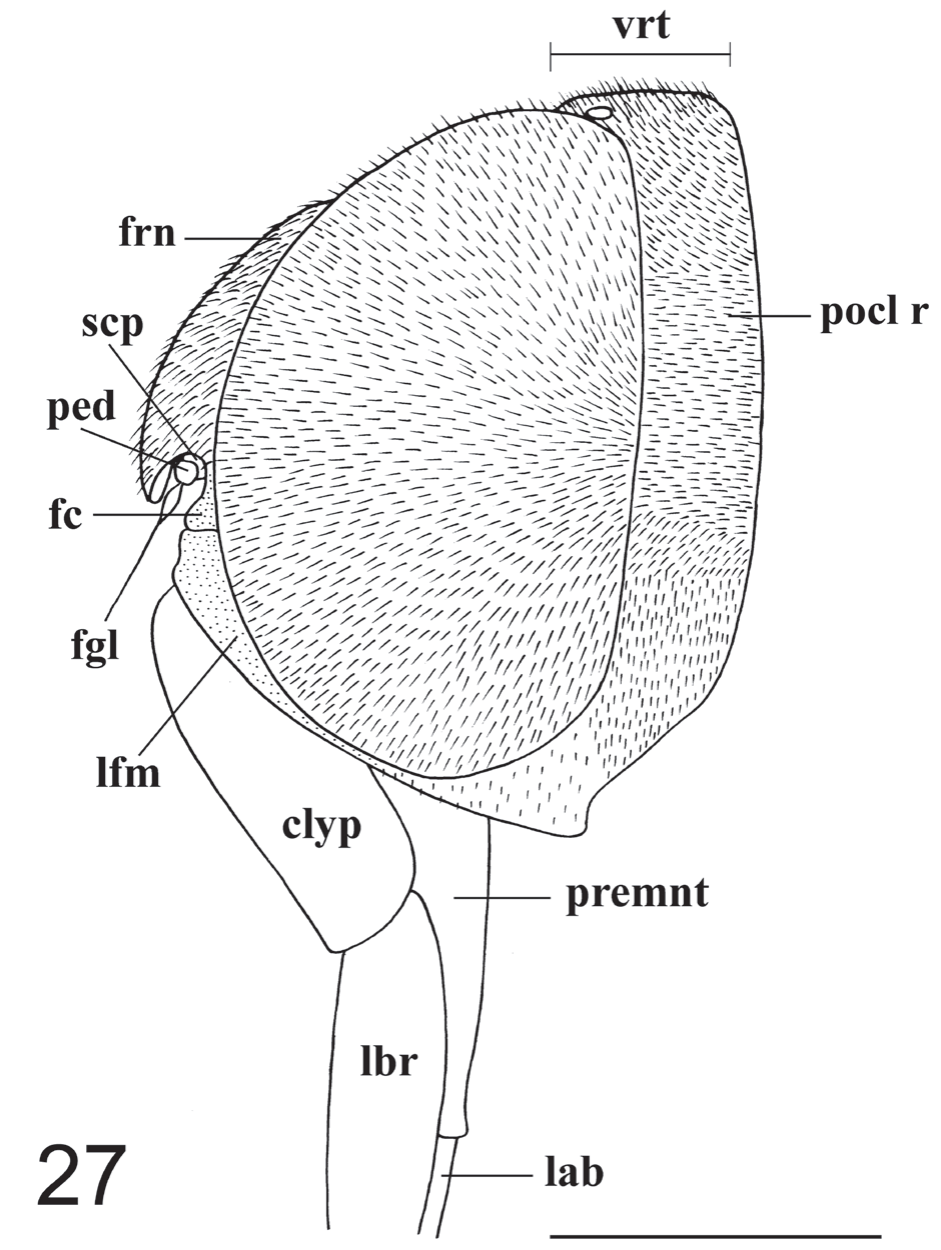
*Philopota conica* Wiedemann, 1830, female, head, lateral view. Scale bar = 1.0 mm. **clyp** clypeus **fc** face **fgl** flagellum **frn** frons **lab** labium **lbr** labrum **lfm** lower facial margin **ped** pedicel **premnt** prementum **pocl r** postocular ridge **scp** scape **vrt** vertex.

**Figure 28.**
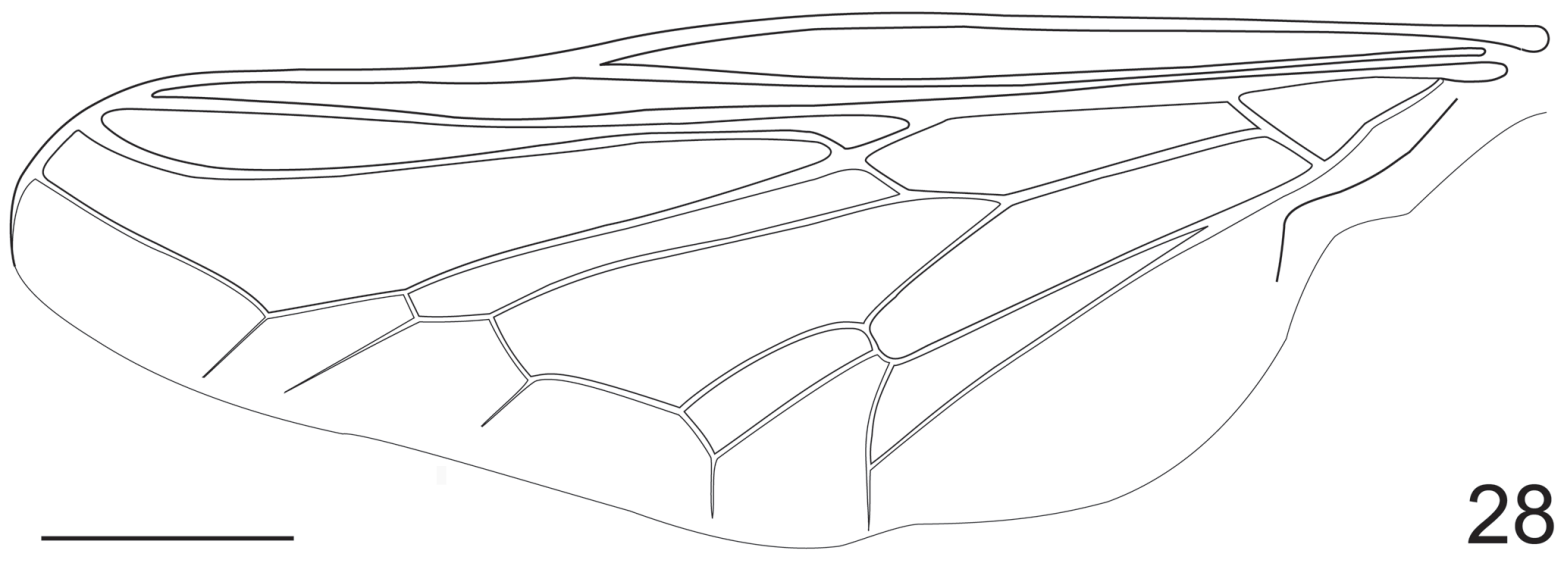
*Pialea antiqua* Schlinger, 1956, female, wing. Scale bar = 1.0 mm.

**Figure 29.**
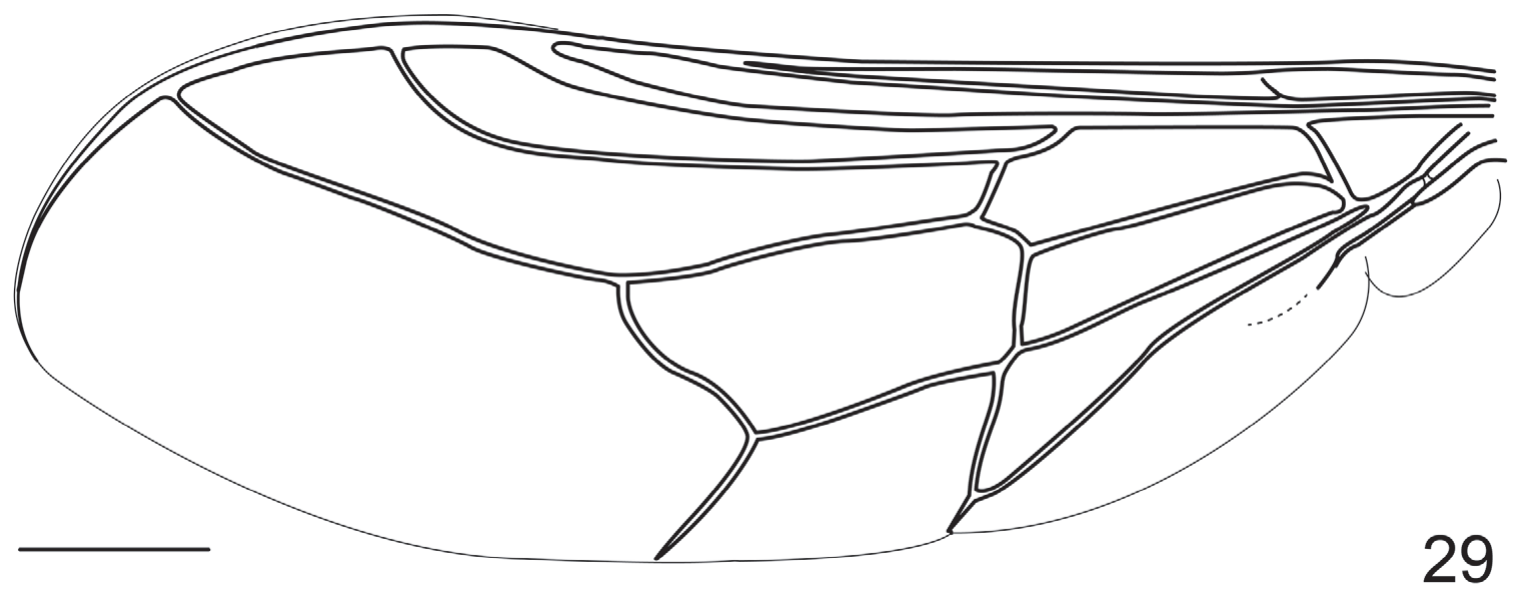
*Pterodontia davisi* Paramonov, 1957, female, wing. Scale bar = 1.0 mm.

**Figure 30.**
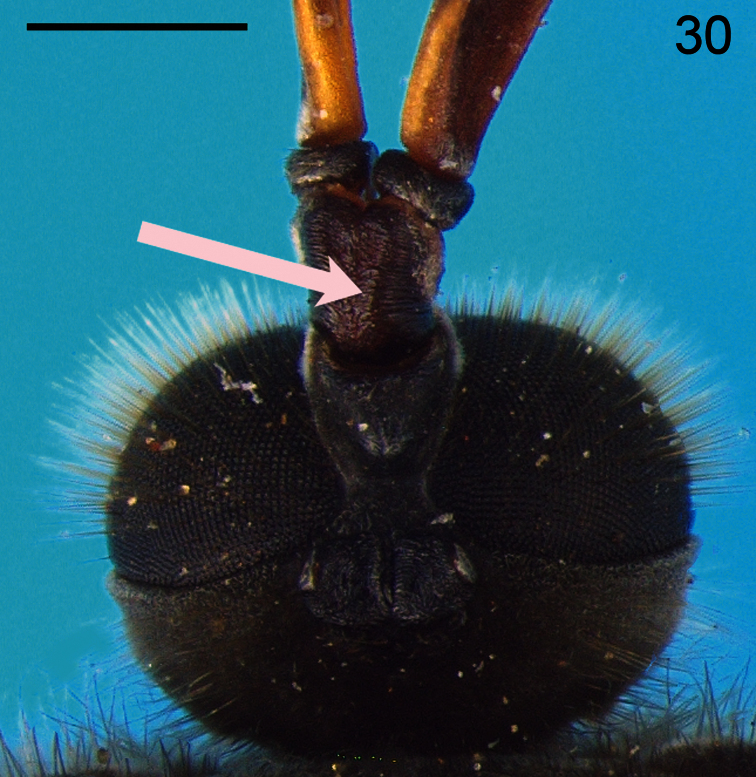
*Pialea lomata* Erichson, 1840, head, dorsal view. Scale bar = 0.5 mm

**Figure 31.**
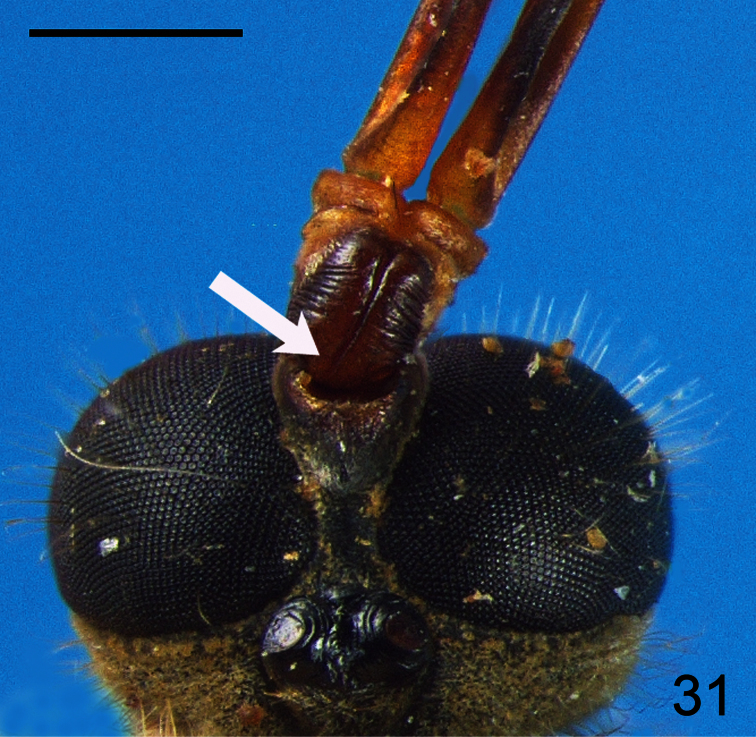
*Pialea antiqua* Schlinger, 1956, head, dorsal view. Scale bar = 0.5 mm.

**Figure 32.**
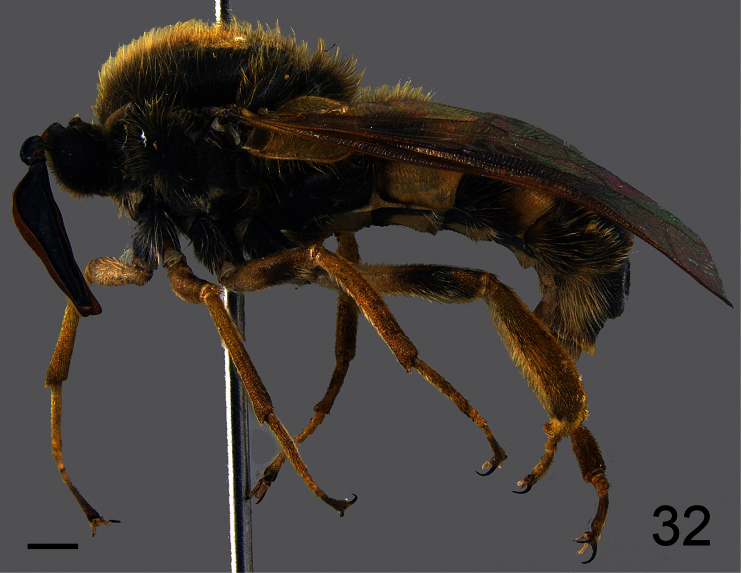
*Pialea antiqua* Schlinger, 1956, male, lateral view. Scale bar = 1.0 mm.

**Figure 33.**
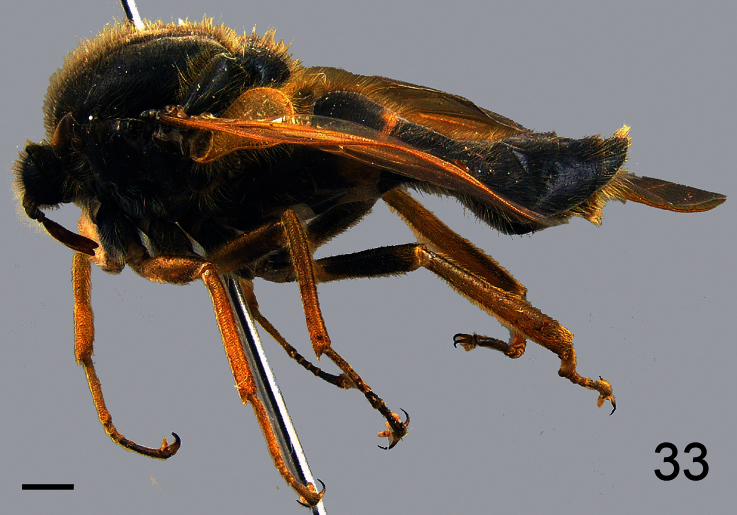
*Pialea antiqua* Schlinger, 1956, female, lateral view. Scale bar = 1.0 mm.

**Figure 34.**
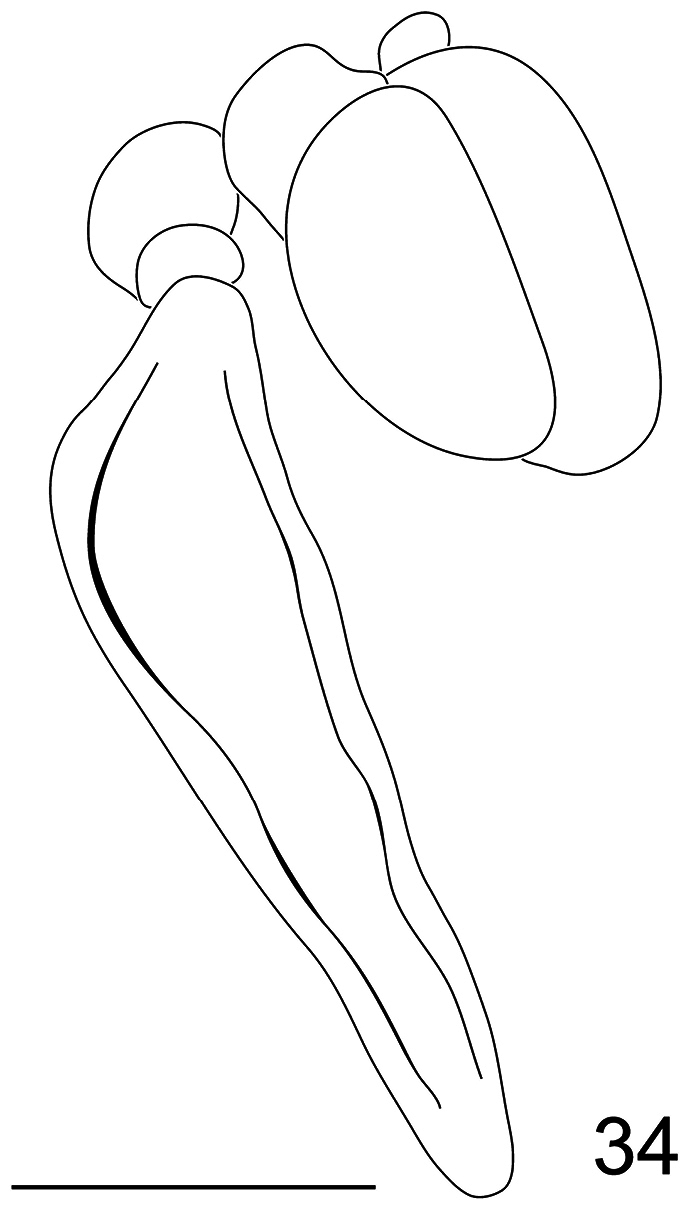
*Pialea antiqua* Schlinger, 1956, male, head, lateral view. Scale bar = 1.0 mm.

**Figure 35.**
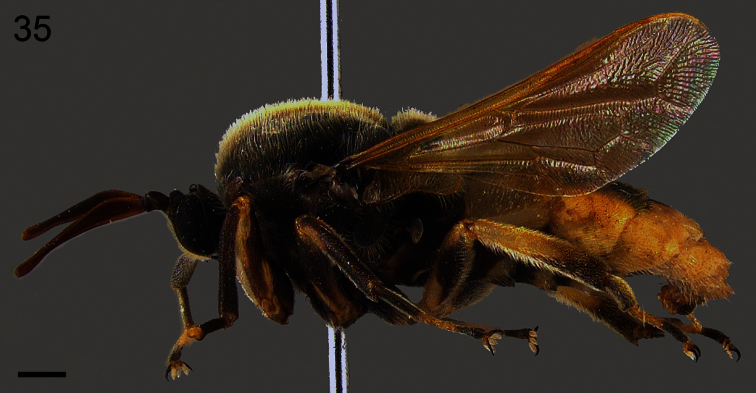
*Pialea lomata* Erichson, 1840, male, lateral view. Scale bar = 1.0 mm.

**Figure 36.**
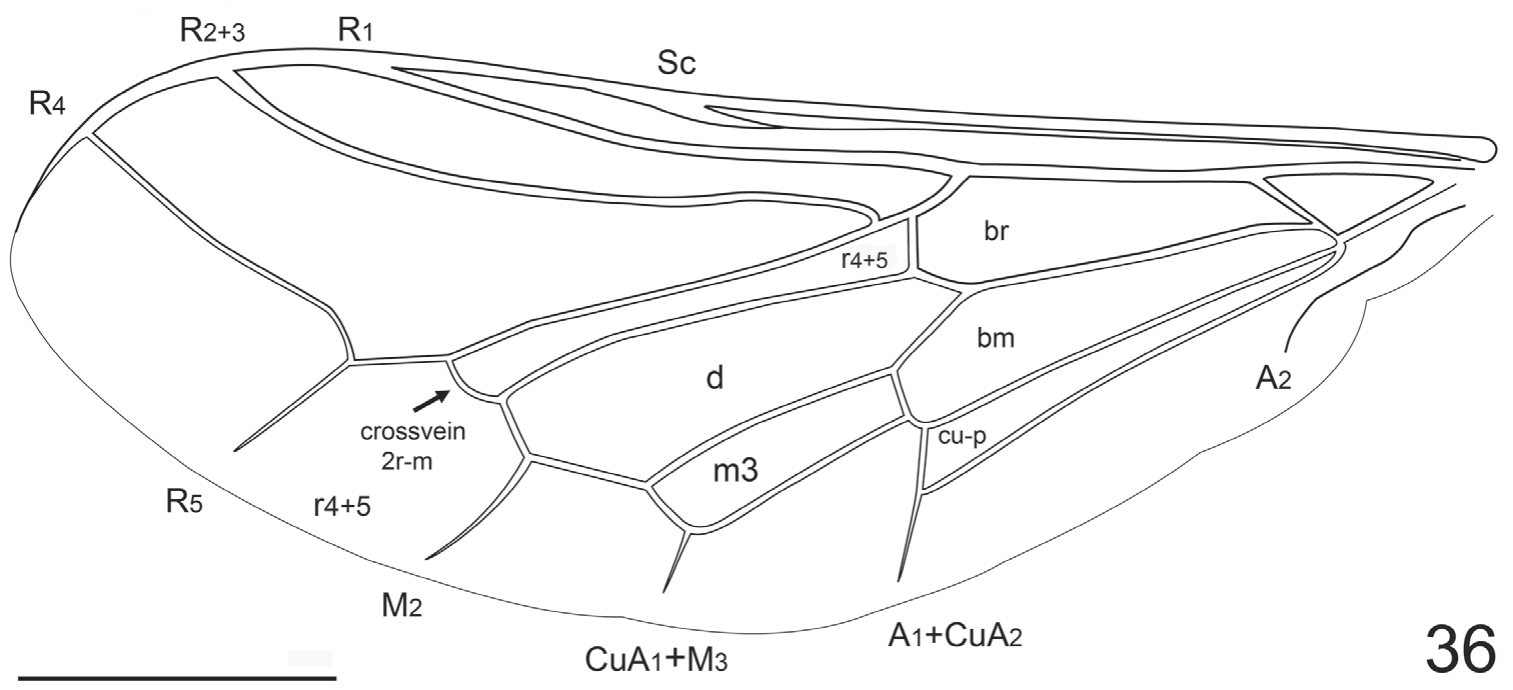
*Pialea lomata* Erichson, 1840, wing. Scale bar = 1.0 mm.

**Figure 37.**
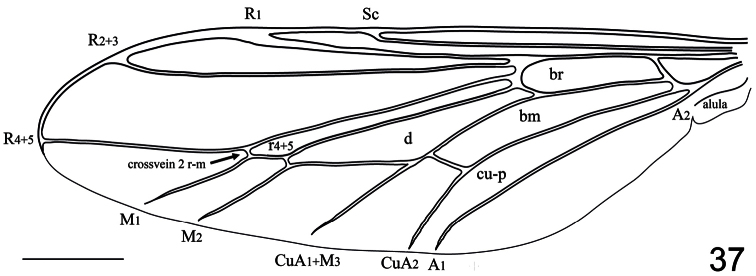
*Villalus chilensis* Cole, 1918, wing. Scale bar = 1.0 mm.

## Supplementary Material

XML Treatment for Sphaerops

XML Treatment for Sphaerops micella

XML Treatment for Sphaerops appendiculata

XML Treatment for Neophilopota

XML Treatment for Neophilopota brevirostris

XML Treatment for Coquena

XML Treatment for Coquena stangei

XML Treatment for Pialea

XML Treatment for Pialea brunea

XML Treatment for Pialea corbiculata
